# Concussion and the Autonomic, Immune, and Endocrine Systems: An Introduction to the Field and a Treatment Framework for Persisting Symptoms

**DOI:** 10.3390/jpm15010033

**Published:** 2025-01-17

**Authors:** Jon L. Pertab, Tricia L. Merkley, Holly Winiarski, Kelly M. J. Cramond, Alex J. Cramond

**Affiliations:** 1 Neurosciences Institute, Intermountain Healthcare, Murray, UT 84107, USA; 2 Department of Psychology and Neuroscience Center, Brigham Young University, Provo, UT 84602, USA; 3 Department of Psychiatry, The University of Texas Southwestern Medical Center, Dallas, TX 75390, USA; 4 Summit Neuropsychology, Reno, NV 89521, USA

**Keywords:** concussion rehabilitation, persisting symptoms post-concussion, autonomic nervous system dysfunction, mild traumatic brain injury, behavioral interventions

## Abstract

A significant proportion of patients who sustain a concussion/mild traumatic brain injury endorse persisting, lingering symptoms. The symptoms associated with concussion are nonspecific, and many other medical conditions present with similar symptoms. Medical conditions that overlap symptomatically with concussion include anxiety, depression, insomnia, chronic pain, chronic fatigue, fibromyalgia, and cervical strain injuries. One of the factors that may account for these similarities is that these conditions all present with disturbances in the optimal functioning of the autonomic nervous system and its intricate interactions with the endocrine system and immune system—the three primary regulatory systems in the body. When clinicians are working with patients presenting with persisting symptoms after concussion, evidence-based treatment options drawn from the literature are limited. We present a framework for the assessment and treatment of persisting symptoms following concussion based on the available evidence (treatment trials), neuroanatomical principles (research into the physiology of concussion), and clinical judgment. We review the research supporting the premise that behavioral interventions designed to stabilize and optimize regulatory systems in the body following injury have the potential to reduce symptoms and improve functioning in patients. Foundational concussion rehabilitation strategies in the areas of sleep stabilization, fatigue management, physical exercise, nutrition, relaxation protocols, and behavioral activation are outlined along with practical strategies for implementing intervention modules with patients.

## 1. Introduction

Approximately 30% of adults and 25% of teenagers report experiencing at least one concussion/mild traumatic brain injury (mTBI) during their lifetime [1,2,3]. While some people experience a rapid resolution of symptoms within a week of sustaining a concussion, others report a protracted symptom course with disruptions in sleep, cognition, emotional stability, and physical symptoms such as headaches, sensory sensitivities, and dizziness [4,5]. Between 30 and 50% of individuals diagnosed with concussion in emergency departments report persisting symptoms at post-injury periods of 6 months or more (see summary in [6] and also [7,8,9,10,11]).

Both the criteria used to diagnose concussion and the relevance of the concussive injury to persisting complaints are topics of controversy among the scientific community [12]. While we will touch on some of these issues in this paper, the primary focus is not to resolve these controversies. Rather, this paper is focused on intervention: practical methods to conceptualize and treat the types of persisting complaints that can occur following concussion. While some authors advocate for a distinction between the terms “concussion” and “mTBI” [13], they are used interchangeably in this paper.

## 2. Concussion and Regulatory Systems in the Body

We previously published a systematic review of the evidence suggesting that dysregulation of the autonomic nervous system following concussion, in both the acute and chronic stages, is one potential factor contributing to protracted symptoms in some patients [6]. Additional reviews since that time further support the presence of a relationship between concussion and autonomic nervous system dysfunction [14,15,16,17,18,19,20].

The autonomic nervous system works in concert with other regulatory systems in the body, including the endocrine (hormonal) system and the immune system [21,22,23,24,25,26]. It is beyond the scope of this paper to describe these relationships in detail, but a brief summary of some core components of these systems is included below at a level that might be relevant to discuss in simplified form with patients. Interested readers are encouraged to consult the following publications for more information about the interaction between traumatic brain injury and the autonomic, endocrine, and immune systems [27,28,29,30,31].

## 3. An Introduction to the Regulatory Systems of the Body

### 3.1. The Autonomic Nervous System

The autonomic nervous system (ANS) supports the automated fine-tuning of organ systems and tissues throughout the body. Autonomic signaling adjusts the activity of organs and tissues to promote an optimal response to changes in the internal or external environment (such as changes in levels of temperature, light exposure, or level of threat). It also regulates organ systems and tissues to respond optimally to changes in behavior (such as eating food, postural changes, cognitive activity, and sexual encounters [21]). The ANS plays a role in regulating blood pressure, body temperature, energy metabolism, the sexual response, gastrointestinal movement and secretion, and many other physical processes. For example, in response to cold temperatures, the autonomic nervous system constricts blood vessels in the periphery, preventing heat loss, and activates a process known as brown adipose tissue thermogenesis, in which glucose and fat molecules are metabolized to generate heat [32,33].

The ANS impacts target organs and structures primarily via a network of nerves that connect the brain to the furthest reaches of the body. The ANS transmits neural signals via its parasympathetic and sympathetic divisions, innervating cardiac muscle, smooth muscle, and various endocrine and exocrine glands, and influencing most tissues and organ systems in the body. (A third division, the enteric system, is not considered in this article, but see [34] for details.) If ANS fibers to an organ are cut, the organ may continue to function, but the organ’s capacity to respond to changing conditions will be compromised [21,35].

In most situations, increased sympathetic signaling prepares the body for exertion/energy expenditure (“fight/flight” reactions when extreme). One example is the rapid response of the sympathetic nervous system to stress. The sympathetic stress response triggers catecholamine release (dopamine, epinephrine/adrenaline, and norepinephrine) from sympathetic nerve terminals to targeted tissues and triggers the adrenal medulla (a small gland on top of the kidneys) to release catecholamines into the bloodstream [36,37,38]. In contrast, parasympathetic activity prepares body tissues and organs for restorative functions (“rest and digest”) and works primarily through the release of acetylcholine from nerve terminals, though other neurotransmitters are also involved [21,39,40]. This cycle between sympathetic activation for energy expenditure demands, followed by withdrawal of expenditure systems when demands pass and activation of parasympathetic restorative and re-energizing systems, promotes physical and psychological wellbeing in individuals and adaptation to the environment [37].

The parasympathetic and sympathetic branches often work in contrasting roles to one another. Upregulation of one branch is typically accompanied by downregulation of the other. A good example of this synchrony is the autonomic control of pupil diameter, which is controlled by the activity of two muscles. The iris sphincter muscle reduces the size of the pupil in response to parasympathetic nervous system activation, and the iris dilator muscle increases pupil size in response to sympathetic activation [41]. The pupillary light reflex (constriction) and the pupillary dark reflex (dilation) reflect the relative dominance of parasympathetic vs. sympathetic activation of the pupillary muscles. An interesting sidenote is that in mTBI research, anomalies in the pupillary light reflex have been found that are associated with the presence of persisting symptoms [42].

As discussed in our previous article [6], the symptoms of concussion are nonspecific. Many other medical conditions that involve anomalies in autonomic nervous system functioning present with post-concussive-type complaints at levels that equal or exceed the level observed in patients with concussion. In part, concussion symptoms may be conceptualized as autonomic dysregulation symptoms. Medical conditions that have large overlaps in symptoms with concussion include chronic pain, chronic stress, depression, anxiety, chronic fatigue syndrome, orthopedic injuries, cervical strain injuries, and sleep disruption. Furthermore, patients with known autonomic dysregulation as their primary diagnosis (such as postural orthostatic tachycardia syndrome) report symptoms similar to those reported by patients with concussion, including fatigue, headaches, nausea, memory dysfunction, attention complaints, brain fog, anxiety, depression, and insomnia (see [6] for a review).

One of the more interesting findings in concussion research relates to increased vulnerability to stressors in people who have had a history of prior concussion. Several preliminary-level studies have displayed that participants with a history of concussion (even asymptomatic samples) tend to have a blunted parasympathetic response to experimental stressors, an exaggerated sympathetic nervous system response to experimental stressors, and a larger cognitive decrement in performance under stressful conditions than do control groups [43,44,45,46,47,48].

Another autonomic function relevant to concussion recovery relates to blood pressure regulation. Concussion may undermine the autonomic control of blood pressure in some patients and contribute to concussive-type symptoms [49]. For example, impaired cerebral blood pressure regulation has been proposed as a key contributor to persistent posttraumatic headache following concussion [50]. Furthermore, impairments in blood pressure control and its consequences may be long lasting. In retired American football players, the level of concussion burden accrued during the playing career correlates with an increased prevalence of hypertension in later life, and these anomalies in blood pressure regulation may increase the risk for late-life cognitive decline [51,52,53]. We discuss the relevance of blood pressure control to post-injury orthostatic intolerance conditions and exercise intolerance in a later section of this paper.

### 3.2. The Immune System

One of the main reasons why the immune system is relevant in concussion physiology relates to the immune system’s inflammation response. The acute inflammation response is the immune system’s initial reaction to harmful stimuli. Immune cells that detect cellular damage or pathogens release various substances, known as inflammatory mediators, that cause the small blood vessels in the affected tissue to dilate and increase their permeability. This increases blood flow, increases fluid infiltration into the damaged tissue (swelling), and increases the transportation of immune system cells from the bloodstream to the injured tissue. This coordinated response transports immune cells to sites where they are needed, removes harmful pathogens, and promotes tissue repair and recovery processes for damaged cells, including cells damaged by brain injury [54]. An optimal immune response involves upregulation of inflammation in the face of threat or injury and resolution of the acute inflammation once the threat has passed [55]. An acute neuroinflammatory response following brain injury is an adaptive reaction that serves to protect neurons from further damage and clear cellular debris [56].

While acute inflammation responses to brain trauma are typically beneficial, poorly regulated inflammation can persist and become chronic and toxic to the brain [57,58]. Chronic inflammation ultimately leads to damage to tissues and organs over time, due in part to ongoing oxidative stress on cells and the paradoxical interference of chronic inflammation on a healthy immune system response [55,59]. The causes of the transition to chronic, low-grade inflammation are not fully understood, but they are thought to be related to a number of factors including environmental toxins, industrial toxins, DNA damage, increasing age, obesity, lack of exercise, poor diet, and poor microbiome diversity [55]. Some authors have discussed the mechanisms by which pre-injury exposure to such factors can reduce resilience to, and healing from, concussive injuries [60,61]).

Chronic neural inflammation can evolve following mTBI, and persistent elevations in neuroinflammatory markers have been found in mTBI samples [62,63]. Markers of persistent neural inflammation are apparent in both active and retired National Football League athletes and in National Collegiate Athletics Association athletes studied in the USA, and these elevated markers of neural inflammation can persist years after the injury [64,65,66]). In a recent systematic review, elevated inflammatory marker levels following mTBI were found to correlate with persisting symptoms [67]. Observations in this area have led some authors to propose the addition of a secondary neuroinflammatory response to the neurometabolic cascade model of concussion [64].

Symptoms such as fatigue, anhedonia, emotional dysregulation, and low mood are typical of chronic neuroinflammation in non-mTBI populations [38,68,69]. Several reviewers have proposed that persistent inflammation could account for persisting symptoms in mTBI patients, including symptoms of irritability, headaches, psychiatric symptoms, sleep disturbances, cognitive difficulties, dizziness, and sensitivity to stress [70,71,72].

Another factor relevant to many mTBI patients is that injuries to other parts of the body that are related to the concussive event (such as musculoskeletal injuries in a motor vehicle accident) can contribute to poor resolution of neuroinflammation. Chronic inflammation in peripheral systems (such as those associated with chronic pain) can lead to inflammation in the brain; this is due to the impact of chronic peripheral inflammation on the integrity of the blood–brain barrier, the functioning of glial cells in the brain and the impact of the immune response on the autonomic nervous system [73,74]. In addition to impacting post-concussive-type complaints, this chronic neuroinflammatory response can be a potent contributor to the neural sensitization that drives chronic pain syndromes and chronic posttraumatic headache symptoms [75,76].

### 3.3. The Endocrine (Hormonal) System

A hormone is a chemical messenger that typically travels through the bloodstream to impact the functioning of tissues and organs throughout the body. There are a network of glands and organs in the body that produce hormones that regulate many functions, including growth, repair, and reproduction. The hormonal systems that are most relevant to the topic of this article are those associated with the hypothalamic–pituitary–adrenal axis (HPA axis), the circadian regulation of the hormones melatonin and cortisol, and other pituitary hormone systems [77,78].

#### 3.3.1. The Hypothalamic–Pituitary–Adrenal Axis (HPA Axis)

The HPA axis is probably most famously known for its role in response to stress. The HPA axis stress response occurs in addition to the rapid response to stress of the sympathetic nervous system described above. Effects along the HPA axis take longer to appear (3 to 5 min) but also last for a longer duration, depending on the stressor duration (potentially multiple hours). Stressors induce signaling in the paraventricular nucleus of the hypothalamus, which triggers the pituitary gland at the base of the brain to release various hormones, including adrenocorticotrophic hormone (ACTH), into the bloodstream. Circulating ACTH causes the adrenal glands (which are located on top of the kidneys) to produce and release hormones known as glucocorticoids, the most prominent being cortisol.

At appropriate levels, cortisol helps the body to deal more effectively with stressful situations by increasing available energy and activating organ systems to meet real or anticipated demands. Cortisol mobilizes energy stores in the liver, fat, and muscles, ultimately increasing available glucose concentrations in the bloodstream. Cortisol release into the bloodstream concurrently provides feedback to the brain to reduce the release of stress-related hormones to maintain an appropriate balance for environmental demands and to end the stress response when the stressor is over [37,79,80]. The healthy activity of the HPA axis under moderate acute stress serves as a temporary boost to energy when required for adaptive responses to the environment [37,81].

The discussion above primarily relates to normal conditions and responses to acute stressors—those that wane after a duration of up to several hours. Individuals can respond very differently to the presence of chronic stressors. This variability is based on genetics, early life experience, environmental conditions, sex, and age. Chronic stress can result in HPA axis dysfunction, which can include chronic basal hypersecretion of cortisol, a sensitized stress response, or adrenal exhaustion, each with their own manifestation dependent on individual factors [36,80].

#### 3.3.2. Circadian Rhythm Hormones

In the absence of stressful conditions, the HPA axis is involved in the normal energy mobilization required for everyday life. One aspect of this relates to the role of the HPA axis in the normal rise of cortisol levels, that peak around the time of normal waking in the morning, and the decline of cortisol levels in the evening. This is one aspect of the circadian hormonal cycle that supports the availability of sufficient energy resources for daily activity. A complementary circadian cycle occurs in the pineal gland’s release of melatonin, with increases in levels prior to and during the typical sleep period and declines prior to waking. This cycle reduces cell metabolism at night and promotes restorative sleep. These circadian cycles are discussed further in the Sleep Module section of this paper.

In mTBI patients, preliminary studies suggest that there tends to be an increase in cortisol levels and cortisol reactivity to exercise stress in the acute phase that persists until at least one month post-injury [82,83]. A recent study found decreased amplitude of diurnal cortisol changes and a blunted cortisol awakening response during the first month post-mTBI; cortisol anomalies were associated with symptom severity and neurocognitive testing results [84]. We were not able to identify any direct research in mTBI related to cortisol levels beyond these timeframes, but longer-term deficiencies in the cortisol releasing hormone ACTH in mTBI samples (see below) suggest that there may be a transition to cortisol deficiencies over time in some patients. Symptoms associated with ACTH deficiency in non-mTBI populations include fatigue, dizziness, depression, anxiety, and orthostatic hypotension [85,86], suggesting that deficiencies in these hormones have the potential to contribute to post-concussive-type symptoms.

#### 3.3.3. Other Pituitary Hormones

Our discussion above has focused primarily on one of the pituitary hormones (ACTH) and its impact on circulating cortisol. The pituitary also releases growth hormone, prolactin, thyroid stimulating hormone, follicle stimulating hormone, luteinizing hormone, oxytocin, and vasopressin. Other endocrine glands throughout the body release other hormones. A full review of the impact of these hormones on physiology is not included in this article, and interested readers can reference the following reviews of this interesting subject [87,88].

In the context of mTBI, chronic pituitary dysfunction is commonly observed following mTBI, with up to 40% of patients displaying anomalies in hormone production, even years after the injury; growth hormone and ACTH deficiencies are the most common findings [89,90,91,92,93,94]. For the purpose of this paper, it is relevant to note that anomalies in pituitary hormones in non-TBI populations include many symptoms that are similar to those associated with concussion, including fatigue, insomnia, impaired cognition, memory loss, difficulty concentrating, and psychological disturbances [86]. A trial of growth hormone replacement therapy in mTBI patients with deficiencies resulted in improved symptoms and changes in brain morphology and connectivity [95]. As the focus of this paper relates to behavioral rehabilitation, the role of pituitary screening and treatment is not covered in detail. However, pituitary screening is recommended for mTBI patients who have persisting symptoms, especially for those who do not respond to the types of intervention efforts that are described in the treatment section of this paper. Pituitary screening and intervention recommendations can be found in the following publications: [85,86,91,96,97,98,99].

## 4. Interaction Effects Between Regulatory Systems in the Body

The interactions between the autonomic, immune, and endocrine systems are so intricate that collectively they may be more appropriately considered a unitary homeostatic system for the body. Medical science has divided this homeostatic process into three systems for convenience and exploration, but influencing any aspect of any one of these three systems impacts the other two. For readers who are interested in learning more about the intricacies of these interactions, the following reviews will be instructive: [23,24,25,26,37].

For the purposes of the present paper, the presence of these intricate interactions is highlighted to alert clinicians to the following opportunity: behavioral interventions targeting the stabilization and optimization of the functioning of any one of the regulatory systems of the body (autonomic, endocrine, or immune) will have complementary benefits to the other systems.

The present paper proposes a treatment framework based primarily on the workings of the autonomic nervous system and its interplay with the endocrine system and the immune system. The rationale for focusing on the stabilization of regulatory body systems in concussion rehabilitation comes from three primary observations:

(a) There is evidence of dysregulation of the autonomic nervous system and other regulatory body systems following concussion.
(b) There is a large overlap of symptoms of concussion with other medical conditions that also impact the regulatory systems of the body.
(c) Therefore, efforts to stabilize the regulatory systems in the body have the potential to reduce symptom burden and improve patient functioning.

## 5. Other Neurophysiology with Potential Relevance in Post-Concussive Complaints

Our focus on the regulatory systems of the body is not intended to imply that these systems are the only relevant processes that have the potential to impact symptoms following concussion. There is a rapidly growing body of research exploring the physiology of concussion. In addition to autonomic, immune system, and endocrine changes, it is probable that additional findings in concussed populations may contribute to the experience of persisting symptoms, including the following:

- White matter anomalies and axonal dysfunction [100,101,102,103,104,105,106,107,108,109],
- Neurochemical and metabolic disruption [110,111,112,113],
- Brain morphology/structural changes [114,115,116,117,118,119],
- Electrophysiological changes [120,121,122,123],
- Cerebral blood flow anomalies, which in part are regulated by autonomic networks [124,125,126,127,128,129,130,131,132,133,134]. See also reviews of cerebral perfusion anomalies associated with various scanning modalities, including single photon emission computed tomography (SPECT) scanning [135,136], transcranial doppler ultrasound [137], functional magnetic resonance imaging blood flow markers [138], and arterial spin labeling MRI [139].

From the rapidly flourishing body of concussion physiology research, we are learning that

- Concussion can involve multiple aspects and markers of neuronal functioning,
- Neuronal anomalies likely evolve and change over time as a patient moves from the acute to chronic stages of recovery (neurotrauma is a process and not a single event) [140],
- The neural physiology of concussion is very complex and likely varies from one individual to another.

There are many interwoven processes that may account for the symptom profile of patients following concussion. The overall picture of the neural impact of concussion is so complex that a clear understanding of the wide array of mechanisms involved may be decades away. Furthermore, it may be several more decades before translational research arrives at clear, evidence-based concussion treatment recommendations that can be reliably applied to individual patients.

In the meantime, this article applies the currently available knowledge to provide a framework that can be readily applied with patients that present for treatment of persisting symptoms following concussion. We recognize that this framework will likely be superseded as research becomes more refined in the future.

This paper advocates for a focus on the impact of concussion on the regulatory systems of the body as a fruitful causative model for treatment planning. While there are likely many other processes involved, the focus is one that allows for (a) a simple model that is easily understood by most patients, (b) hopeful expectations in patients, as these systems are malleable, and (c) practical application of several behavioral intervention modules that have the potential to improve patient functioning and reduce symptom burden. These points will be discussed along with specific treatment strategies in later sections of this paper.

## 6. The Status of Current Treatment Research in Concussion

While there has been some commendable progress in research that directly evaluates specific treatment strategies for concussion, controlled treatment trials are scant in the literature and of limited quality for both adults and children (see reviews [141,142,143,144,145,146,147,148,149,150,151,152,153,154]).

While there is a desire among clinicians for research-based guidelines to help patients reduce persisting symptoms following concussion, there is also limited treatment research to base these guidelines on, resulting in some confusion in clinicians seeking to tailor treatments for specific patients who are seeking help [155].

### 6.1. In the Absence of Well-Developed Treatment Research, What Do Clinicians Draw on to Guide Treatment Decisions?

In the preface to the American Academy of Neurology *Clinical Practice Guideline Process Manual*, the authors outline **three pillars** that clinicians employ to make treatment decisions [156]: *“**Evidence** is only one source of knowledge that clinicians use to make decisions. The other two sources are established principles—for example, the **neuroanatomic principles** that enable neurologists to know precisely that a patient has a lesion in the lateral medulla just by examining the patient—and **judgment**, the intuitive sense clinicians rely on to help them decide what to do when there is uncertainty (emphasis added).”*

Given that the **evidence** (controlled treatment trials) in the area of concussion rehabilitation is limited but valuable and instructive, this paper proposes a number of treatment modules for concussion patients based on the **neuroanatomic principles** that we are currently aware of and provides some guidance based on clinical experience regarding how clinicians can use **clinical judgment** to tailor treatment priorities to the individual patients that they encounter.

The typical patients we have treated in our clinics are people in the general population who present at emergency departments with injuries sustained in motor vehicle accidents, falls, sports, and assaults, and who commonly have failed initial management strategies in the primary care setting. They usually enter our clinics between 3 and 12 months post-injury. The framework discussed in this paper outlines what rehabilitation clinicians can do to help patients understand the etiology of their persisting complaints and what strategies they can employ to reduce persisting symptoms.

We recognize that rehabilitation resources vary widely by community, and specialized equipment and training are not available in some areas and clinics. This paper focuses on a foundation program that can be implemented in situations where there are limited resources and referral options. This program can be effectively implemented by a wide range of rehabilitation professionals who have appropriate specialized training and interest in the area of concussion rehabilitation, including clinical neuropsychologists, rehabilitation psychologists, psychological therapists, physical therapists, speech and language pathologists, occupational therapists, athletic trainers, neurologists, sports medicine physicians, primary care physicians, physical medicine and rehabilitation physicians, and other rehabilitation therapists.

While we are focusing on patients with persisting symptoms months after their injury, a briefer intervention based on the principles discussed in this paper would be appropriate for patients in the acute stages post-injury to promote recovery. We also recognize that some people (particularly those without premorbid and/or comorbid stressors) recover quickly and well following a concussion, without formal interventions.

### 6.2. Comment Regarding the Use of Medications in Concussion Rehabilitation

This paper does not comprehensively review pharmaceutical interventions with concussion patients, but rather, behavioral interventions that can be readily applied by a wide variety of rehabilitation professionals. The research base for the use of medications following concussion is very limited, with few quality studies available. Some reviewers propose that the research is so weak that pharmaceutical approaches cannot be recommended for clinical practice [142,157,158]. Despite the lack of evidence, the “off-label” use of pharmaceuticals in concussion management is common [159], even in pediatric patients [160,161], and may have some utility in the individual patient presenting for treatment [161,162,163]. Our only comment regarding medications is to advocate for caution in considering the costs and benefits of medication use in mTBI patients. There are several reasons why caution is recommended:

(a) Some medications commonly used in concussion management have the potential for the unintended side effect of hampering the functioning of the autonomic nervous system, potentially impeding overall recovery even though some symptom relief may occur. Classes of medication where caution is indicated include SNRI and (to a lesser extent) SSRI medications, tricyclic antidepressants, antihistamines, antipsychotics, and beta blockers [163,164,165,166].
(b) Some classes of medication present risks in the context of mTBI recovery. For example, some sleep-inducing medications (such as benzodiazepines and atypical gamma-aminobutyric acid (GABA) agonists such as zolpidem) could potentially prolong recovery from TBI, as there is evidence that they may adversely affect cognition and neuroplasticity [167]; analgesic overuse can precipitate headache symptoms [168]; and use of opiates following TBI can increase neural inflammation, impair plasticity, decrease myelin repair, increase neurodegeneration, and present risks for dependency in a vulnerable population [169].
(c) Some patients are involved in occupational or athletic settings where drug testing is employed, which precludes participation for people using certain classes of medication (prescribed or otherwise). It is important for prescribers to be aware of restrictions as part of a cautious approach. For example, in the USA, the National Collegiate Athletic Association, which regulates student sports in university-aged athletes, includes the following banned medication classes: stimulants, beta blockers, various hormones, and beta-2 agonists.

These findings, suggesting limited evidence and potential complications from the use of medications in mTBI, suggest that where possible, clinicians should initially attempt to manage symptoms via behavioral rehabilitation strategies such as those described in this paper rather than primarily relying on medication as a first-line intervention. In cases where behavioral interventions do not lead to symptom relief, pharmaceutical treatments and other medical interventions, administered and closely monitored by an experienced physician, are an appropriate next step.

## 7. Implications for Clinical Assessment and Treatment Rationale for Patients

In the discussion above, we have identified several medical conditions that have extensive symptom overlap with the symptoms associated with concussion. This suggests that clinical assessment of post-concussive-type complaints should include a holistic assessment that includes a focus on comorbid factors with the potential to contribute to such complaints, including the following:

- Review of injury history details and associated acute features at a level that enables the clinician to diagnose concussion based on current diagnostic criteria. This can help avoid iatrogenic impacts associated with misdiagnosis of concussion when one has not occurred.
- Physical and psychosocial stressors.
- Psychiatric history, including screens for depression, posttraumatic stress symptoms, and other anxiety symptoms.
- Chronic pain status, including screening for cervical strain symptoms, and history of pain/fatigue conditions.
- Sleep quality, including sleep apnea screening.
- Orthostatic intolerance symptom history, including vulnerability to presyncope and syncope.
- Pre-injury and post-injury physical exercise involvement and tolerance.

As a side note, this paper is focused on post-acute care of patients who are typically several months post-injury. Appropriate evaluation in the acute phase (first week) includes a physician or other appropriately licensed and trained clinician completing a physical evaluation, for which guidelines and assessment forms have been previously published [170].

### 7.1. Brief Comment on Benign Paroxysmal Positional Vertigo

A two-page brief screening questionnaire covering several of the vulnerability areas listed above is available from the primary author on request. It also includes a screen for benign paroxysmal positional vertigo, which is usually a quickly treatable inner ear disorder that occurs in approximately 25% of concussion patient samples presenting for treatment [171,172]. Early referral for canalith repositioning procedure treatment is recommended for patients who screen positive for this form of vertigo. Other dizziness-type symptoms tend to resolve in most patients with the foundation program described below.

### 7.2. Brief Comment on Comorbid Pain Conditions

The patient’s pain status is also important to evaluate, and any patient screening positive for cervical strain or other types of non-headache chronic pain typically will warrant an early referral for physical therapy evaluation and treatment. The symptoms of cervical strain are indistinguishable from those of concussion [173]. Surprisingly, chronic pain conditions are more common in mTBI than in more severe forms of brain injury [174]. The reasons for this are unclear, but several theories have been proposed, including mTBI-related neuroinflammation, increased vulnerability to neuropathic pain, white matter vulnerability to enhanced pain perception, upregulation of chemical pain signaling, and downregulation of descending pain modulation circuitry [175,176,177,178]. Regardless of the reasons for increased pain in mTBI, pain is known to dysregulate the functioning of the autonomic, endocrine, and immune systems and has the potential to be a significant barrier to stabilization and treatment of post-concussive-type complaints if not addressed [179,180,181,182,183,184].

### 7.3. Brief Comment on Comorbid Psychological Features

Concussion increases the risk for the development of anxiety and depression; this includes exacerbation of pre-injury conditions and new-onset disorders in those with no pre-injury history of mental health diagnoses [185,186,187,188]. Initial explorations of the reasons why this is the case include the possibility that acute differences in the activation and structure of cortical emotional processing circuits following mTBI may contribute to the development of emotional-related symptoms [189]. In chronic samples, the number of previous concussions correlates with psychiatric symptoms, anomalies in the limbic system, and indicators of chronic inflammation impacting neurotoxic metabolites that have been implicated as risk factors for psychiatric disturbance [118,190,191]. In addition to these injury-related neuropathological processes, psychosocial stressors—such as physical and cognitive symptoms interfering with participation in activities such as work, school, athletic, or family roles—are common in the aftermath of concussion. These stressors, combined with experiences such as misattribution of symptoms to untreatable factors, can also be potent contributors to psychological distress following injury [192,193].

Uninjured populations who present with anxiety and depressive disorders also have high levels of post-concussive-type symptoms [194]. Effectively addressing psychological distress is an important clinical target to avoid the extra burden of psychological distress on post-concussive-type complaints. The treatment modules described below will support emotional regulation in patients, whether or not they have access to immediate psychological therapy. In fact, the treatment modules for concussion discussed below contain many features that are central to “lifestyle interventions” for mental health conditions in the general population that also serve to promote brain health in general [195,196,197,198,199,200,201].

In addition to the beneficial mental health impacts of the treatment modules described in this paper, the emotional support, empathy, hope installation, and validation that many physicians and therapists provide (even when they do not have formal psychological training) has the potential to be beneficial to health outcomes and psychological stability [202,203]. (However, see the comment below on potential iatrogenic effects.)

While many symptoms of psychological distress will likely spontaneously improve by the application of treatment modules for concussion discussed in this paper without additional psychological interventions, a significant exception relates to the presence of traumatic reactions associated with the injury mechanism. These include symptoms of acute stress disorder (or in cases of longer duration, posttraumatic stress disorder), including re-experiencing symptoms, avoidance and numbing, and hyperarousal symptoms that are associated with the event. Screening for such symptoms in the initial evaluation is important. In one study of over 1000 mTBI patients, the prevalence of PTSD symptoms at 3 months and 6 months post-injury was approximately 20% [204]. When patients screen positive for PTSD-type symptoms, early referral for psychological therapy is indicated. One study has displayed significant benefits from a structured cognitive behavioral therapy intervention with concussion patients presenting with symptoms of acute stress disorder [205].

For patients who do not experience some improvement in psychological symptoms during the course of the intervention modules described below, or in patients where psychological features appear to be a barrier to them engaging in the intervention effectively, a referral for psychological therapy can also be considered.

### 7.4. The Treatment Rationale

Once the assessment is complete and the factors potentially contributing to persisting symptoms have been identified, the clinical formulation can be shared with the patient in an educational format, often in the initial appointment. This education might typically include the following:

- A basic overview of the autonomic nervous system and its functions,
- A brief discussion regarding the potential impact of concussion on the autonomic nervous system,
- Information regarding the overlap of concussion symptoms with other conditions that impact autonomic functioning,
- An individualized formulation outlining the factors that are potentially contributing to their post-concussive-type complaints,
- A discussion of the treatment rationale and initial focus.

Education regarding the interaction of the autonomic nervous system with the endocrine and immune systems is often completed in subsequent appointments to avoid overwhelming the patient with more information than they can effectively consume. It is appropriate to provide written summaries, diagrams, and handouts of the information at a level appropriate to the individual patient (or family members if working with young children) to support comprehension and retention.

**Figure 1:**
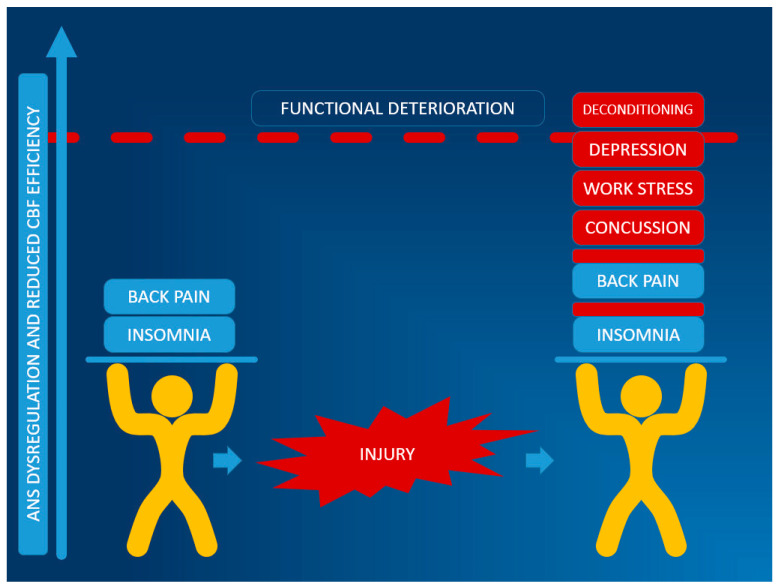
A simplified graphical representation of factors that may be impacting autonomic nervous system functioning pre-injury and post-injury for a specific individual.

The educational process can be enhanced by graphically presenting features relevant to the specific patient formulation. An example in Figure 1 can be tailored to individual patients of various ages and circumstances and can be accompanied by a script similar to the following: “*Many of us experience circumstances like periods of poor sleep and chronic stress that interfere with the smooth functioning of the autonomic nervous system. If these challenges are not too numerous or intense, we do not usually experience a major change in our ability to go to work/school or take care of responsibilities in the home. Sometimes following an injury, the pre-injury factors such as stress and poor sleep can be worsened by the effects of the injury, the concussion itself can destabilize the autonomic nervous system, and often people experience other injury related conditions that further impact the smooth functioning of the autonomic nervous system. In your case, the following factors are potentially impacting your overall symptoms [list the factors specific to the individual case]. People can often get away with one or two moderate factors impacting their autonomic nervous system without large impacts on their daily life. However, if these factors are numerous or severe enough, people reach a point where the symptoms they experience limit their ability to complete tasks. The treatment program that we are recommending has several foundation modules to improve the smooth functioning of the autonomic nervous system.”*

The benefit of presenting this model to the patient and educating them about the autonomic nervous system’s role is that it is easily framed as a message of hope. Hope has been shown to improve outcomes in many physical and psychiatric conditions [206]. Furthermore, expectation effects and fear avoidance processes have been proposed as potential factors contributing to prolonged recovery [207,208,209,210,211]. Patients may be able to do little to address some of the neural impacts of concussion directly (such as the cell death and axonal shearing described in Giza and Hovda’s “neurometabolic cascade” model [111,212,213]). The hopeful message is that in most patients, there are multiple malleable factors (that are likely contributing to their symptoms) that can be treated. Additionally, there are several behavioral interventions that have the potential to enhance or optimize the functioning of the autonomic nervous system and other regulatory systems in the body.

The following sections outline the rationale for specific treatment intervention modules that serve the function of stabilizing autonomic, endocrine, and immune system functioning following concussion and reducing symptom burden. The research in many of these areas is vast, and we have focused on information that we might convey to patients in simplified form as a rationale for the treatment recommendations of each section.

## 8. Sleep Module

### 8.1. Neuroanatomical Principles: Sleep and Concussion

Sleep disturbances are common after concussion, with up to 40% of patients experiencing long-term sleep disruption [214,215,216,217,218]. The persistence of poor sleep following injury is one of the strongest predictors of poor recovery outcomes in adults and children [217,219,220,221,222,223,224]. Sleep–wake disturbances may actually be more common following mTBI than in more severe forms of TBI [225,226,227]. Higher rates of pain in mTBI samples compared with more severe TBI samples may partially account for this [174,228].

There is anecdotal evidence via patient reports that many patients with persisting post-concussion symptoms present with a history of initial hypersomnolence following injury. This could potentially contribute to inconsistent patterns of sleeping and napping during the day, which could in turn contribute to developing insomnia due to dysregulation of circadian rhythms [229]. Maladaptive sleep habits and dysfunctional cognitions related to sleep may serve to increase arousal and performance anxiety related to sleep, which act in opposition to the state of relaxation necessary to induce sleep [230].

Sleep difficulties in concussion patients can include insomnia; increased need for sleep; poor quality, fragmented sleep; and excessive daytime sleepiness [231,232]. Disruptions in sleep following concussion not only relate to the duration of sleep but also less-efficient sleep patterns, shorter rapid eye movement onset latencies, longer sleep onset latencies, reductions in deep slow-wave sleep stages, and reduced rapid eye movement sleep [233,234].

There are several neuroanatomical principles that suggest that an early focus on stabilizing sleep is relevant for patients with persisting symptoms of concussion. These relate to a) the impact of sleep on the regulatory systems in the body, b) the role of sleep in cleaning waste toxins from the brain, and c) the role of sleep in restorative/healing processes. The following paragraphs briefly outline the research that is relevant to these areas.

### 8.2. Sleep and Regulatory Systems in the Body

Sleep has bidirectional interactions with regulatory systems in the body. Suboptimal sleep can compromise the optimal functioning of the autonomic, endocrine, and immune systems; likewise, disruptions to the regulatory systems in the body have the potential to disrupt the circadian hormonal signaling required to maintain a healthy sleep profile [235,236,237,238,239]. The occurrence of sleep disturbances following concussion could be caused in part by neuroinflammatory processes following the trauma, and these sleep disturbances could, in turn, result in neuroinflammatory-related tissue damage [75].

The sympathetic branch of the autonomic nervous system is often elevated when sleep is of poor quality or short duration in non-injured populations [240,241]. This hyperarousal may, in part, be related to the observation that extended reduction in sleep is a metabolic stressor and can result in neuron loss in the locus coeruleus, which is an important structure in autonomic regulation, arousal regulation, pain regulation, emotional regulation, and stress reactivity [242,243,244,245].

The relevance of sleep and its relationship to the regulatory systems has also been considered in the context of concussion. The acute immune response to concussion has been hypothesized to undermine the circadian functioning that regulates the sleep–wake cycle and attenuate the beneficial cortisol surges that support energy during the waking hours [29,231]. Several summaries have outlined how sleep changes following concussion can impact the functioning of the autonomic, hormonal, and immune systems [246,247].

The symptoms of sleep deprivation have a large overlap with the symptoms of concussion [248,249,250]. It is possible that one common factor accounting for these similar symptoms is that both sleep and concussion can undermine the optimal functioning of the autonomic nervous system and other regulatory systems in the body. Thus, effective treatment of sleep has the potential to improve concussive-type symptoms.

### 8.3. Sleep and the Glymphatic System

Concussion is associated with an acute “metabolic cascade” of cellular injury, which can result in the accumulation of extracellular proteins and other waste products in the fluid surrounding cells in the brain, including tau and amyloid proteins and their byproducts [111,251]. Furthermore, several studies suggest that energy utilization in some regions of the brain can remain elevated following concussion, and this can persist for months following the initial injury; hypoactivation in other regions is noted [133,252,253,254,255,256,257,258,259]. Whenever energy is used in the brain, waste products are produced, such as carbon dioxide, lactate, and proteins including amyloid-ß and tau proteins [260]. In humans, elevations in neural waste products such as tau and amyloid proteins are found in military personnel many months after their concussion history [113,261,262].

One of the primary systems that cleans these toxins from extracellular brain tissue is the glymphatic system [260]. In brief, this system involves an extracellular fluid pulse/pressure wave that is generated by the pulsatile action of the arteries in the brain. The pressure wave flows from the spaces surrounding the arteries, through the fluid between the brain cell matrix, to the spaces surrounding the venous system in the brain. As the wave travels through the brain, it collects waste products and transfers them to systems that excrete them from the body. The glymphatic system also delivers helpful compounds like glucose, lipids, amino acids, and neurotransmitters to brain cells [263,264,265]. Research has identified the central role of sleep in this cleaning process, with the efficacy of the system being approximately 95% less efficient during the wake vs. sleep state in animal models [266,267]. Animal model research also suggests that the efficiency of the glymphatic system may be compromised following concussion [268].

Improving sleep to support the glymphatic efficacy of cleaning waste products from the brain following concussion can be seen as a primary clinical target a) due to the importance of cleaning out extracellular byproducts of the initial injury (the so-called “neurometabolic cascade”), b) to reduce the buildup of waste products generated by longer-term increases in neural energy demands, and c) research in humans suggests that long-term changes in glymphatic markers can occur following concussion and impact symptom recovery [269]. One behavioral strategy to improve these glymphatic risk factors following concussion is to implement behavioral interventions to improve sleep.

### 8.4. Sleep and the Restorative Systems in the Brain

Each cell in the brain converts glucose into cellular brain energy in the form of adenosine triphosphate (ATP). ATP is stored in brain cells and used to fuel brain activity [270]. During the quiescent state of sleep, there is a surge in adenosine triphosphate production in the brain, and this energy is used for the anabolic restorative processes that occur during the sleep period [271]. These restorative processes (including promotion of synaptic plasticity and protein synthesis) are of particular import in the context of the potential cellular damage associated with concussion.

### 8.5. Evidence: Sleep Treatment in Concussion

There are multiple modalities that have shown promise for treating insomnia in the general population [272]. The recently published European Insomnia Guideline advocates that all patients with insomnia, with or without comorbid medical or mental health problems, be offered cognitive behavioral therapy for insomnia (CBT-I) as the initial treatment [273]. Several reviews document the benefits of CBT-I in both psychiatric and medical populations [274,275,276,277,278,279].

The components of CBT-I have been summarized in several books and articles [280,281,282,283,284,285]. The treatment is typically provided as a multicomponent treatment, including tracking using a sleep diary, psychoeducation, relaxation therapy, sleep restriction therapy, stimulus control therapy, and several cognitive strategies to address unhelpful thinking patterns that can interfere with sleep. Treatment is usually brief, between four and eight sessions, and in many patients may take only a small portion of a 50 min treatment session, allowing concurrent treatment of other treatment targets.

There is a small body of evidence that supports the use of CBT-I with patients who have sleep difficulties following a concussion [286]. Uncontrolled treatment trials combining behavioral and pharmacological modalities (including melatonin and prazosin in concussion patients with comorbid PTSD) have shown initial promise [217,287].

Three randomized controlled trials of CBT-I following concussion have been completed. Malarkey et al. [288] conducted a randomized clinical trial of CBT-I delivered in an internet-only (no clinician involvement) format for six weeks and compared the outcomes with an internet-based sleep education program. Of the 106 originally enrolled, 41 adult veterans with a mTBI history completed the study. Significant differences on the Insomnia Severity Index were in favor of the treatment group at the end of the intervention but were not significant at the 3-month follow-up, largely due to improvements in sleep quality of the control group during the follow-up period. Ludwig et al. [289] conducted a randomized controlled trial of clinician-administered CBT-I with a treatment phase of six weeks (via Zoom or phone) using a staggered intervention with a wait list condition. Participants included 32 adults with a concussion history of mixed etiology and with duration since injury of at least one month. For those completing the intervention, 81% displayed clinically relevant improvements on the Insomnia Severity Index (seven-point drop or more). Tomfohr-Madsen et al. [290] conducted a randomized controlled trial of 6 weeks of CBT-I with 24 concussed adolescents between 12 and 18 years old who had persisting post-concussive symptoms. Participants in the intervention group had significant improvements in insomnia ratings and a modest improvement in post-concussive symptoms following the intervention and at one-month follow-up compared with treatment as usual. (See also Lah et al. [291] for a case series of CBT-I in adolescent concussion patients.)

Other evidence relevant to CBT-I in concussion comes from mixed-group trials that include patients with mTBI combined with more severe brain injury groups. A scoping review of CBT-I after mixed-severity traumatic brain injury concluded that “*…CBT-I may improve sleep quality, reduce insomnia symptoms, and decrease depression and anxiety, fatigue, and symptoms severity in individuals with TBI”* (see also [292,293]). The case series described by Ouellet and Morin [294] is an example of a study that provides support for the potential of CBT-I to be beneficial in concussion rehabilitation. They report on 11 participants with traumatic brain injury history ranging from mild to severe. A standard 8-week treatment of CBT-I was completed, including stimulus control, sleep restriction, cognitive restructuring, sleep hygiene education, and fatigue management. Clinically and statistically significant reductions in total wake time and improved sleep efficiency were apparent for 8 of the 11 participants. An average reduction of 53.9% in total wake time was observed across participants from pre- to post-treatment, and sleep efficiency improved from pretreatment (77.2%) to post-treatment (87.9%), and from post-treatment to the 3-month follow-up (90.9%).

In conclusion, early research focused specifically on mTBI populations provides support for implementing CBT-I symptoms in individuals with a history of concussion. Other areas of insomnia research, including samples drawn from the general population, psychiatric patients, non-TBI medical conditions, and in mixed traumatic brain injury samples, also support the potential for behavioral sleep treatment benefits in individuals with a history of concussion.

### 8.6. Clinical Judgment: Sleep Treatment in Concussion

Due to both the potential benefits of improving sleep and the risks for protracted concussion-type symptoms when patients struggle with insomnia following injury, sleep is typically the first area of focus in the stepwise intervention program for concussion patients that we are describing in this paper.

Sleep tracking is a core component of the intervention [295] and is often discussed and implemented toward the end of the assessment session so that tracking information is available at the first treatment appointment. Sleep tracking for at least a week or two is even recommended for patients who do not endorse prominent difficulties with sleep in the assessment. Our experience is that a significant number of these satisfied sleepers have sleep habits that are suboptimal for achieving the full rehabilitation benefits of quality sleep. Sleep advice in the absence of formal tracking (for example, sleep hygiene education alone) seems like it has the potential to improve sleep, but research has shown that it typically does not. Sleep hygiene discussions alone (even though they may include a description of many components of a formal CBT-I program) are so impotent in promoting change that they are sometimes used as a control condition in sleep intervention research [273,296,297,298,299].

During the first treatment session, the sleep diary/sleep log can be reviewed, and the clinician can decide if sleep intervention is warranted. For prominent sleep dysregulation, either in timing of sleep (such as a three-hour variance in retiring or rise times across the course of the baseline) or duration of sleep (such as less than six hours average), a formal CBT-I program is typically warranted.

**Figure 2:**
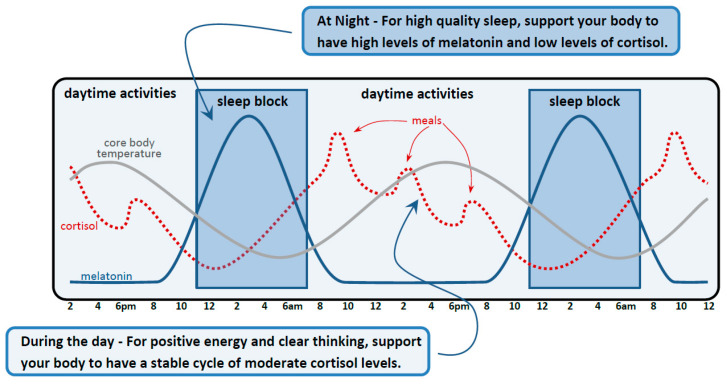
A simplified depiction of circadian rhythm hormones that impact sleep and daytime energy levels.

For patients with less severe sleep disruption, stabilization can often be achieved by a less rigorous single-session intervention focused primarily on the enhancement of the circadian hormonal cycle. The initial portion of the intervention is education. This psychoeducation portion related to sleep is an ideal opportunity to discuss the role of stabilization of the three main regulatory systems in the body, their interconnectedness, and their relevance to concussion rehabilitation. We would typically review handouts regarding the importance of bolstering the endocrine circadian rhythm cycle due to its central role in promoting restorative sleep in the night and boosting energy and alertness during the day (a relevant consideration considering the prevalence of fatigue in patients with post-mTBI complaints). A simplified model focusing primarily on the hormones melatonin and cortisol is shared with patients (see Figure 2). The regulation of these hormones is primarily based on light exposure; light exposure in the morning (full spectrum but particularly light that includes blue-green tones) signals to the brain that it is daytime and synchronizes time regulation centers in the hypothalamus to release melatonin in the evening to promote sleep. Artificial light exposure in the evening can hamper the production of natural melatonin [300]. The effects of light exposure on melatonin are mirrored in an opposite direction on the HPA axis control of diurnal variation in cortisol levels (bright blue-green light increases cortisol, and dim warm lighting decreases it [81]).

Specific recommendations are made based on the large body of research that has been conducted regarding circadian rhythms (see [301,302,303,304,305,306]). These recommendations include the following:

- Set up a regular sleep block of about 8 h in duration. Be consistent in your bed and wake times.
- Get at least 30 min of direct light (preferably outside) sometime in the morning. (As an aside, in addition to supporting sleep, sunlight exposure may also improve vitamin D levels, which can reduce chronic inflammation, upregulate neurotrophic factors, and regulate oxidative stress following brain injury [307].)
- Avoid bright lights and blue-green tinted light at night by doing the following:
  - Closing curtains and dimming lights a few hours before your bedtime.
  - Using red-colored bulbs in your lamps at night.
  - Installing a warm-colored night light in places like bathrooms and hallways
  - Avoiding screens for an hour or two before bed (alternatively, experiment with using blue-green light-blocking glasses—the most effective are the 99% blockers, which are deep orange or red in color).
- Avoid stressful activities and have a wind-down period for at least an hour before your bedtime.
- Use the following activities to support your mind and body to unwind (a list of activities is generated in discussion with the patient).

If patients do not respond to these base recommendations, a formal cognitive behavioral therapy intervention for sleep is initiated (see references above for program description). It is noted that there have been several studies in concussion showing benefits from light therapy devices, but our typical focus in the context of limited resources is to advocate for the use of natural, unfiltered outdoor sunlight; light therapy devices remain an option where resources allow (see [308,309,310,311]).

Based on research previously reviewed, we would consider sleep stabilization so important in the rehabilitation process that we may delay or postpone discussion of other modules of treatment until some progress in this area is achieved. Time spent in motivational interviewing strategies [312] to support patients to engage in increased rigor to a sleep program is time well spent.

In the rare case that we meet with concussion patients in the acute phase, where hypersomnolence is more prominent, or encounter post-acute patients (beyond one month post-injury) where they are sleeping in excess of 10 h a day, we recommend strategies to stabilize the circadian cycle. Specific recommendations include restricting sleep to a night block not to exceed 10 h (working gradually toward 8 h) and a midday block typically not to exceed 1 h; we also encourage patients to avoid resting in bed during the daytime. These measures support normalization of the sleep–wake cycle and help reduce the development of sleep fragmentation and insomnia [294].

It should be noted that sleep recommendations are sometimes very challenging for patients to implement in cases where migraine and prominent associated photophobia are present. In the case of severe migraine, it is often appropriate to retire to a dark bedroom and rest. We advocate for supporting patients toward a growth process where we share ideal practices and support them to implement strategies as their circumstances allow. Reviewing tracking sheets with patients is an effective way to explore the costs and benefits of episodes of low adherence from a collaborative and experimental framework that respects the autonomy of the patient.

## 9. Fatigue Module

### 9.1. Neuroanatomical Principles: Fatigue and Concussion

The concept of fatigue in neurologic conditions can include physical components (tiredness/exhaustion), psychological features (lack of motivation, reduced future orientation), and cognitive components (difficulties with concentration, sustained attention, increased mental effort) [313]. When considering the construct of fatigue in general, up to 60% of people report increased fatigue after mTBI [214,218,314]. Some patients describe that post-TBI fatigue is often associated with the feeling of “hitting a wall,” where further engagement in mental or physical activity is severely limited due to associated cognitive dysfunction, sensory overstimulation, pain, and sleepiness [315].

One of the factors that may contribute to fatigue following brain injury is reduced cognitive control. This can result in higher neural energy expenditure compared with non-injured individuals to compensate for reduced cognitive efficiency. In simple terms, the brain must work harder than usual to complete tasks following injury. This finding is relatively robust in moderate to severe traumatic brain injury; mixed findings in mTBI research suggest that only a subpopulation may present with increased activation patterns following injury, or only under certain conditions [316]. In general, this effect is more likely in symptomatic individuals and varies depending on the time since injury [124,127,253,316,317,318,319,320].

In the general population, the experience of fatigue correlates with an array of alterations in the functioning of the autonomic, hormonal, and immune systems [321]. Fatigue is thought to be one of the core manifestations of autonomic nervous system dysregulation. Adults and children with fatigue in the general population and in chronic fatigue syndrome tend to have increased sympathetic activation and reduced parasympathetic activation [322].

Fatigue is one of the first and most common symptoms associated with an activated immune system. In patients with cancer or hepatitis C who are administered pro-inflammatory treatments, symptoms of fatigue have rapid onset in approximately 80% of patients [323]. This suggests that fatigue is very sensitive to the effects of cytokines. Cytokines are immune system signaling proteins that help control inflammation in the body. Multiple studies indicate atypical cytokine levels in both the acute and chronic phases following mTBI; the direction of change is variable, with some finding increases and some decreases compared with controls (see systematic review [67]). In one recent study, elevated cytokine levels in the acute phase following mTBI predicted which patients developed persisting post-concussive-type symptoms [324]. It is probable that immune system alterations may contribute to developing symptoms of fatigue following mTBI.

The immune system cytokines also modulate endocrine systems such as the hypothalamic–pituitary–adrenal axis, activating the release of corticotropin-releasing hormone, adrenocorticotropic hormone, and cortisol. As previously mentioned in the sleep section, circulating cortisol is one of the hormones that is involved in promoting positive energy for daytime activity as part of the circadian hormonal cycle. The experience of fatigue has been associated with elevated cortisol levels and depressed cortisol levels, but most reliably with anomalies in diurnal variability—the cycle of cortisol levels across the course of the 24-h day [325,326,327,328,329]. Early research indicates that patients with mTBI tend to have elevated levels of cortisol in the acute and subacute phase of recovery [82,83], and this could be a further factor driving fatigue in this population due to its impact on the circadian cycles described previously.

In populations without a history of concussion, both overexertion and underexertion are associated with fatigue and cognitive difficulties in a range of clinical and non-clinical populations [330,331,332,333].

#### 9.1.1. The Risks of Underexertion and Overexertion

In the situation of underexertion, long-term bed rest represents the more severe end of the spectrum. Difficulties with postural balance, cognition, and emotions are associated with bed rest, and the negative impact of bed rest is magnified in older individuals [334,335,336]. Bed rest also weakens the entrainment of the circadian system to the 24-h day [337]. The deconditioning caused by bed rest can be functionally debilitating in patients attempting to return to normal activities [338].

Supporting mTBI patients to avoid the negative cardiovascular, musculoskeletal, circadian, and baroreflex impacts of excessive rest is an important clinical opportunity, as there is large clinical overlap between the symptoms of deconditioning and the symptoms associated with concussion. Increased sedentary activity levels are apparent following concussion in pediatric patients, and the impact of this on recovery is a relevant consideration [339].

There are several fields of research that are instructive regarding the impact of overexertion on the regulatory systems in the body. One of these areas is research exploring the physical impact of athlete overexertion/overtraining. A host of complaints can accompany excessive physical exertion in athletes, including many that overlap with post-concussive-type complaints, including fatigue, low mood, insomnia, irritability, concentration decline, and anxiety [340]. Overexertion in athletes is associated with anomalies in the autonomic, endocrine, and immune systems, including decreased heart rate variability associated with autonomic dysregulation and increased sympathetic tone, alterations in hypothalamic–pituitary–adrenal axis hormones such as increased cortisol and reduced adrenal sensitivity, and immune system dysfunction in the form of inflammation and cytokine release [340,341,342].

While the findings are less consistent, similar autonomic, endocrine, and immune system anomalies are apparent in some general population samples involved in research into the phenomenon of burnout—a condition associated with chronic occupational and lifestyle overexertion in non-athletes [343,344,345,346,347,348]. Symptoms associated with overexertion in burnout also have large overlap with post-concussive symptoms, including emotional exhaustion, anxiety, irritability, sleep impairment, physical fatigue, and cognitive fatigue [343].

In summary, patients with concussion display anomalies in neural, autonomic, hormonal, and immune system functioning. In some patients, these anomalies may contribute to the experience of fatigue. Furthermore, behavioral patterns observed in the general population and in various clinical conditions suggest that both overexertion and underexertion are associated with the experience of fatigue and other post-concussive-like symptoms. This highlights the importance of supporting patients to avoid the risks associated with these extremes of activity and finding an intermediate zone of activity where the optimization of regulatory systems is fostered. This intermediate zone is a principle applied to a wide variety of medical interventions and in medical research is often referred to as the Goldilocks zone. This is in reference to the 19th-century English fairy tale “Goldilocks and the Three Bears.” In one aspect of this story, Goldilocks identifies porridge that is not “too hot,” not “too cold,” but “just right.”

The following section reviews the development of research into developing exertional management strategies that have the goal of being “just right” for patients post-concussion.

### 9.2. Evidence: Fatigue and Concussion

Historically, clinicians have recommended fatigue management as a core component of recovery following TBI. The following quote from John Hilton in 1867 [349] is one of the earliest recommendations in modern medicine that advocates for fatigue management following concussion.

*“In concussion of the brain, as soon as the blow which strikes the skull has caused the symptoms of concussion, the physical disturbance of the brain, whatever it may be, has been produced… Such a disturbed brain is defective—if not in structure, certainly in its vital endowments, and is therefore unequal to even its ordinary duties. It recovers itself slowly; it then soon becomes fatigued from use; and if claims are made upon it too soon after the injury—that is, before structural and physical integrity is reacquired—the patient is very likely to suffer from a serious disease of the brain. Cerebral exercise or mental occupation should always in such cases be short of fatigue. The brain requires absence from occupation, or rest, for its complete recovery, and this should be in proportion to the severity and duration of the symptoms of concussion…”*

Specific advice regarding the balance between rest and activity following mTBI has varied widely over time, from moderate symptom-limited activity to more extreme prescription of activity limitations such as “bed rest,” which became popular in some countries throughout the 20th and early 21st centuries [350,351,352]. As research in this area developed, a number of notable reviews over the past decade have highlighted the potential detrimental impact of extreme rest prescription in concussion rehabilitation, and more moderate activity restrictions have been promoted [352,353,354,355,356,357,358,359,360]. Unfortunately, the overprescription of rest continues to be prevalent in healthcare, and it is prescribed to a large percentage of patients with mTBI, with negative impacts on functional outcomes [361].

The research available at this point in time recommends a more moderate approach for fatigue management programs, reducing activity to a level that does not significantly exacerbate symptoms but not beyond that. This stance of moderation is supported by observational studies in pediatric concussion populations; those with low or high activity levels post-injury tend to perform more poorly on follow-up assessments than patients with moderate activity [355,362]. In adults, persisting symptoms were more pronounced in mTBI patients with sedentary behavior vs. those that engaged in moderate levels of physical activity [363]. As a side note, in another observational study, moderate levels of screen time in the first 10 days post-injury correlated with relatively less severe post-concussive-type symptoms during the first 30 days post-injury compared with high or low users of screens [364,365]).

Fatigue following mTBI is a significant predictor of limitations in social functioning, physical functioning, quality of life, activity levels, and return to employment status [314,366,367]. Despite the importance of addressing fatigue in mTBI, there are very few published behavioral rehabilitation studies that directly target fatigue.

Ali et al. [368] conducted a systematic review of treatment studies in all-severity TBI that included fatigue as an outcome measure even if fatigue was not specifically targeted in the treatment modality. (Earlier reviews are noted: [369,370].) In fact, only one intervention reviewed directly targeted fatigue. This intervention employed education and problem-solving therapy related to managing post-TBI fatigue in a sample of 41 TBI participants randomized to an intervention or attention control group [371]. The intervention aimed to build awareness, identify fatigue triggers, and adopt energy conservation and problem-solving compensatory strategies to manage fatigue. Findings were positive for impact on fatigue with small to moderate effect sizes following the 8-week program. Positive results were also reported in a follow-up study [372].

### 9.3. Clinical Judgment: Fatigue and Concussion

In clinical practice with post-acute concussion patients, clinicians will encounter patients who overexert and patients who underexert. Both of these extremes have risks for protracted recovery.

For patients who underexert in the form of sleeping excessively (more than 9 h a day) and strictly limiting activity, a reconditioning program that includes normalizing sleep schedules and gradually increasing daily activity is indicated. Patients who sleep too long and do very little during the day are at risk for physiologic deterioration over time. In the general population, excessive sleep duration is a risk factor for autonomic dysfunction, orthostatic intolerance, increased immune system markers for inflammation, and phase delays in circadian hormonal rhythms [373,374]. Sedentary behavior patterns are also risk factors for autonomic, endocrine, and immune system anomalies [375,376,377,378].

Patients who overexert following concussion tend to present with a cyclic pattern of high levels of activity, which triggers exacerbation of symptoms, which in turn forces a period of low levels of activity until symptoms subside. This “roller coaster” of up and down levels of activity can occur within the day (for example, several hours of overexertion period followed by several hours of exacerbated symptoms forcing low activity) or between days (for example a “crash” of several days after a particularly demanding day of excessive activity that exacerbates symptoms). This “roller coaster” pattern has been described as a “boom-and-bust” pattern by other authors exploring interventions in the field of concussion [379], but we are not aware of any systematic explorations of this pattern in the research literature.

The practicalities of addressing fatigue and implementing a fatigue management program to avoid overexertion and underexertion patterns following concussion are relatively similar regardless of the pattern patients originally present with. Both patients who overexert and underexert will benefit from a structured approach that promotes (a) the individualized identification of a Goldilocks zone that establishes a maximum level of activity that does not significantly exacerbate symptoms and (b) gradual increases in activity over time as tolerance for activity improves. We acknowledge that this advice is remarkably similar to that proffered by John Hilton in the 1800s, as noted in the quote above—*“in proportion to the severity and duration of the symptoms”.*

Dorothy Gronwall, a neuropsychologist from New Zealand, and her colleague, neurosurgeon Phillip Wrightson, established the country’s first concussion clinic at Auckland Hospital in the 1970s. Based on a series of research studies and decades of clinical experience in the 1970s through 1990s, they developed a program of concussion rehabilitation that closely reflects the program that we are advocating for in the present publication. Their advice remains consistent with subsequent research into the treatment and physiology of concussion that is reviewed in this paper, including recommendations for sleep intervention, relaxation training, “gentle exercise,” counseling and support for patients with psychological features, and a focus on fatigue management strategies. In a book summarizing their clinical experience and research insights [380], they advocate for a fatigue management program that includes the following features:

- Evaluation of activity tolerance.
- Development of a daily schedule with activity periods, rest periods, and a structure for specific tasks to work on and a breakdown of those tasks into manageable portions.
- Adjusting the timing of demanding activities to periods where patients are typically most energized.
- Gradual increases in activity and work hours as dictated by patient symptoms.
- Education of the patient so that they are aware that progress is rarely linear; episodic setbacks are expected, even in cases where an overall improving trajectory is apparent.
- Emotional support to manage the stresses associated with setbacks and limitations.

Many of these features have been adopted by speech therapists who manage fatigue in TBI populations [367,381].

#### 9.3.1. The Importance of Tracking

As with sleep interventions, it is our experience that when transitioning from sleep interventions to a fatigue management program, tracking is a powerful tool. Once sleep is stabilized, transitioning from a sleep log to an activity/energy log is an appropriate next step. In practice, for most patients, we encourage a week-to-a-page tracking sheet/log where patients can record their activities and levels of energy (on a 10-point scale, where 10 = high energy, 1 = drained/“wired tired”) at regular intervals over the course of the day. Activities completed during the morning, afternoon, and evening periods are also documented in the log. Hours of sleep secured the previous night are also recorded to monitor stability in this important variable and its associated impact on fatigue. On average, patients tend to overestimate their activity levels and display more sedentary behaviors than they self-report [382]. Despite likely inaccuracies, tracking provides data that are typically better than no data and reliance on the patient’s memory for activities and their relative impacts during the week.

The tracking sheet can be modified for younger children by a detailed discussion with parents/caregivers of behavioral observations that they typically see when their child is energized and the types of behaviors that are observed when their child is fatigued. They can develop a 10-point scale based on these end points to obtain a rough idea of the fatigue level of their child and can share this scale with other caregivers and teachers if the child is beginning a return-to-school program. A simplified log where parents record activities and energy estimates during three sections over the course of the day (morning, afternoon, and evening) will provide data that are useful for developing fatigue management recommendations. For an excellent discussion of how younger children tend to present symptomatically following concussion and a list of observable behaviors to be aware of, see Beauchamp et al. [383] and Dupont et al. [384].

In the absence of specific research guidance and as a general rule of thumb, we advise patients that if overall symptoms increase up to 2 points on a 10-point scale, that is probably reasonable; however, any increase beyond this should lead to initiating either a rest period or a reduced intensity in the activity they are engaged in. This is the general rule of thumb that we use for both cognitive and physical exertion, and it avoids the risks that some patients present with if they are given advice to “stop if there is any increase in symptoms.” This may be appropriate in cases where patients tend to underestimate and under-monitor their symptoms, but in most cases, the “roughly 2-point difference” advice avoids hyperfocusing on symptoms and over-monitoring for slight increases that may be incidental or generated secondary to the intensive monitoring process itself. This 2-point threshold strategy is consistent with other exertional recommendations post-concussion [385].

It is important to highlight to patients that recovery can come in one of two forms: improvements in presenting symptoms and/or increased tolerance for activity. Sometimes patients bemoan the fact that symptoms are not markedly improving, when they are in fact making progress in activity tolerance during a fatigue management program. In these cases, highlighting the increases in tolerance for activity as evidence of recovery is important to promoting and maintaining hope and positive expectation effects [386]. It is our experience that if patients learn skills and strategies to manage their energy and are diligent in tracking trends, their capacity for increased daily activity usually increases over time.

While there is no well-developed research-based program to increase activity and manage fatigue following concussion, the recommendations above are consistent with the known physiology of fatigue and the available research into recovery patterns following concussion. A highly individualized approach that respects the autonomy and lived experience of the patient, adapts to individual patient circumstances and preferences, and includes quality data gathering to inform the patient’s decision making, increases the probability of clinical improvement.

## 10. Exercise Module

### 10.1. Neuroanatomical Principles: Exercise and Concussion

There is a solid body of research regarding the benefits of exercise on brain functioning in the general population (see reviews in [75,387,388,389,390,391,392,393,394,395,396,397]). These benefits include the following:

- Upregulation of endocrine functioning, including regulation of cortisol levels,
- Improved balance between the sympathetic and parasympathetic branches of the autonomic nervous system,
- Reduced neuroinflammation and upregulation of neuroprotective mechanisms,
- Improved brain blood flow regulation (cerebrovascular autoregulation),
- Improved mood, emotional regulation, and reduced physical pain, possibly due to synergistic effects of exercise-induced increases in the concentrations of dopamine, serotonin, endogenous opioids, and endogenous endocannabinoids,
- Increased brain-derived neurotrophic factor, which promotes neurogenesis (neuron production) and synaptic plasticity, learning, and memory,
- Increased vascular endothelial growth factor, which promotes proliferation of blood vessels in the brain (angiogenesis) and protects against neuronal cell death (apoptosis),
- Increased insulin growth factor, which fosters increased vasculature and neuron production (neurogenesis) in locations of the brain such as the hippocampus,
- Upregulation of mitochondrial density and production,
- Reduced oxidative stress,
- Reduced cognitive decline.

Noting the beneficial impacts of exercise on brain functioning in general, several authors have proposed that exercise is a promising intervention in brain injury recovery [398,399,400,401].

Exercise is also relevant to the gut microbiome, a topic that is discussed in more detail in the nutrition section of this paper. Exercise in non-injured populations promotes helpful diversity in the gut microbiome (eubiosis), which serves to resist the chronic inflammation that can develop following injury [402]. Furthermore, the deterioration of gut microbiome health secondary to unhealthy eating patterns is mitigated to some extent by the beneficial effects of exercise on the gut microbiome and immune system regulation [389,397].

Sleep disruption following concussion is another factor that may be mitigated by exercise. Regular exercise increases slow-wave sleep and total sleep time, possibly due to the impact of exercise on improving parasympathetic tone, reducing anxiety, increasing nocturnal melatonin, and reducing inflammation [403,404]. In a study of concussion in adolescents, those who exercised more than 150 min a week in the post-acute phase had improved sleep quality compared with those who exercised less than 150 min [405].

Exercise intolerance (symptom exacerbation in the face of exercise challenge) is a common symptom among populations with protracted recovery following concussion and is hypothesized to be a manifestation of cerebral blood flow anomalies related to autonomic dysfunction, though there is only a limited number of studies that have directly tested this relationship [16,406,407]. Emerging evidence from small trials in mTBI patients displays that exercise training has the potential to normalize atypical cerebral blood flow and structural connectivity measures in people with post-concussive symptoms and improve overall symptom profile [408,409,410].

### 10.2. Evidence: Exercise and Concussion

Some of the most influential research regarding exercise in concussion rehabilitation has emerged from the University of Buffalo Concussion Clinic, initially led by John Leddy, MD and Barry Willer, PhD. Their review [411] summarizes their efforts to develop an individualized “Systematic Evaluation of Exercise Tolerance After Concussion.” In part, the test employs the Balke Treadmill Test protocol [412]. Heart rate, symptoms, and perceived exertion are monitored while patients exercise on a treadmill with progressive increases in difficulty every minute. Options for stationary bicycle adaptations have also been developed [413], and full protocol instruction manuals are easily searchable online.

The heart rate at which patients terminate the exercise test described above is typically used to calculate a target heart rate for daily exercise, which is defined as 80 to 90% of the termination heart rate. Patients are instructed to exercise at the target heart rate for 6 to 7 days a week for 20 to 30 min. Exercise sessions are ended early if overall symptoms increase 2 points or more on a 10-point scale, and the patient resumes the next day. Ideally, the patient is re-tested on the treadmill test every 2 to 3 weeks to establish a new target heart rate. The goal is to increase heart rate over time by gradually increasing the intensity of training sessions with “graduated sub-symptom threshold exercise” until the patient can exercise at an estimated 80% of maximum heart rate (maximum is operationalized to 220 – age) without symptom exacerbation. This target represents the end of the treatment program.

In an early controlled study of this approach in adults with persisting symptoms (between 1 and 71 months post-injury), approximately 72% of those that successfully completed the exercise rehabilitation program (41 of 57) returned to full functioning in daily activities. Notably, exercise benefited those that were initially intolerant to exercise and also those patients who could exercise to 80% of their estimated maximal heart rate without symptom exacerbation at initial testing [414].

Several recent systematic reviews have explored the impact of graded aerobic exercise on symptoms following mTBI, with generally promising results from early clinical trials at both the acute and chronic post-injury phases of recovery [143,144,358,415,416,417,418].

High-quality randomized clinical trials with large numbers of participants have not yet been completed, and questions regarding the optimal timing of exercise post-injury and the frequency, duration, and intensity of exercise prescription remain unanswered [143,417]. The balance of evidence suggests that initiating such programs within the first week, or at any point beyond that, tends to promote recovery in most patients [143,387,417]. For example, in a large observational prospective multicenter cohort study of over 2,413 children and adolescents with concussion, early participation in physical exercise reduced the prevalence of persisting symptoms at one month post-injury (25% vs. 43%, [419]). In a recent randomized controlled study of six weeks of aerobic training in a mTBI sample that averaged more than two years post-injury, improvements in post-concussion symptoms, quality of life, depression, anxiety, and fatigue were apparent in the aerobic exercise group after six weeks of training [420].

It is important to note that prescribing exercise in the absence of supervised/guided monitoring and tracking has tended to lead to disappointing results for post-concussive complaints and compliance in child, adolescent, and adult studies [417,421,422,423,424]. We note the similarities of this observation with poor outcomes from sleep hygiene instructions compared with programs that involve structured monitoring and tracking (see above).

Some researchers are becoming inventive in modifying the standard Buffalo exercise protocol:

- Some include interval training components, based on research in general populations, which display some advantages of interval training over continuous training for aerobic capacity, cardiovascular health, mitochondrial biogenesis, and vascular function [425,426,427,428]. Wu et al. [429] found modest benefits for adding blood flow restriction and body-cooling apparatus to a moderate-intensity interval training program for persisting concussion symptoms in adults.
- Evaluation using a march-in-place protocol of increasing metronome speed rather than treadmill or bicycle assessment was used by Haider et al. [430] to evaluate exercise tolerance in a military concussion sample. Adding a graduated aerobic exercise program based on the marching test results to the recovery process reduced average recovery time from 24 days to 17 days.
- Use of lower body negative pressure during aerobic exercise and a supine tilt during cycling to prolong exercise tolerance post-concussion [431].
- Combined aerobic–resistance exercises (light weight circuit) have been proposed but not yet tested [432].

#### 10.2.1. Comment Regarding Orthostatic Intolerance and Concussion

There is a growing awareness that in patients with persisting symptoms following concussion, there is a high prevalence of orthostatic intolerance complaints [433,434,435,436]. Some of the primary manifestations of orthostatic intolerance are lightheaded or dizzy symptoms triggered by either extended standing, high temperatures (such as showering), or posture changes such as shortly after standing up or bending over. In some patients, these presyncope-type symptoms may be interspersed with full syncope/fainting episodes.

Asking patients about the presence of presyncope or syncope episodes is an important part of the assessment process, as orthostatic intolerance symptoms can mimic symptoms of concussion [437,438]. If patients endorse frequent difficulties in these areas (multiple times a week), a referral to a physician who is experienced in these conditions (such as electrophysiology–cardiology or an experienced rehabilitation physician) is warranted to screen for heart conditions that may be contributory. More typically, these difficulties are identified as related to altered autonomic control of the baroreflex and peripheral vasculature, with associated reductions in brain blood pressure as a primary driver of symptoms [49]. Contributions from vestibular system dysfunction are also likely in some patients [439]. In both cases, specialized physical therapy intervention is indicated as the primary treatment option. The method by which orthostatic intolerance is evaluated is important, as early research suggests that standard orthostatic vital signs as a standalone test may have poor sensitivity to orthostatic intolerance following concussion [440].

In the case of orthostatic intolerance, there are multiple components to a behavioral intervention that have been described elsewhere [440,441]. One of the primary components is a graduated exercise program that includes both aerobic and strength-training components; while the aerobic component is very similar to the concussion exercise intervention described above, recumbent or semi-recumbent training is often incorporated as a first step [442,443,444]. Given the prevalence of orthostatic intolerance conditions in populations with persisting symptoms following concussion, it is important for physical therapists working in this area to evaluate for and adapt exercise programs for concussion patients with concurrent orthostatic intolerance.

### 10.3. Clinical Judgment: Exercise and Concussion

Ideally, all patients with persisting symptoms following concussion would have access to a specially trained physical therapist/physiotherapist who could complete orthostatic tolerance testing and systematic exercise testing to determine the optimal initial prescription of exercise and regular appointments to make adjustments over the treatment period. Physical therapists who are trained in these interventions and have an additional skill base in rehabilitation for cervical strain injury, vestibular rehabilitation, cardiorespiratory anomalies, or pain management in general are particularly valuable, as many patients with persisting symptoms following concussion have concurrent difficulties in these areas, and these difficulties are also improved by appropriate exercise [356,445,446,447,448,449,450].

For most patients who remain symptomatic in the chronic phase, initiating a graduated sub-symptom-threshold aerobic exercise program seems reasonable and is the most supported advice given the current literature base [144]. Clinicians who are not physicians or trained physiotherapists should have the patient consult with their primary care provider or another doctor involved in their care to ensure that there are no comorbid medical conditions that would make engaging in aerobic training contraindicated. A number of contraindications for engaging in exercise testing and treatment are listed in the “Buffalo Concussion Treadmill Test (BCTT)—Instruction Manual” (available online), and in most patients, clearance for exercise from a physician is appropriate.

In locations where specialized physical therapy services are not available, clinicians can recommend an exercise program to patients based on the available research, but they should be cautious in their approach. While supervised exercise programs following concussion have low risk for adverse events [451], there are risks associated with too much exercise in terms of the recovery trajectory. For example, in one study, higher volumes of vigorous activity in the first 3 days post-injury (72 min/day) were associated with longer recovery times in youths with sports concussions compared with those with moderate volumes (36.9 min/day) [452]. Too little exercise also has limited efficacy. For example, adolescents who responded to an exercise program following concussion (defined as symptom-free recovery in under a month—average of 19 days) exercised, on average, 4.4 days a week for 49.0 min each session; non-responders (average recovery 53 days) exercised, on average, 3.1 days a week for 30.4 min measured by actigraphy; both groups were instructed to exercise 100 min a week for 4 weeks, but without supervision or guidance their engagement in exercise was variable [453]. These types of studies highlight the importance of finding the patient’s individual “Goldilocks zone”—a zone where exercise prescription is “just right” for the individual patient.

In terms of prescribing exercise, currently, an individualized approach using the exercise testing described above and adjustments to the exercise prescription dependent on tolerance appears to be the option with the most research support. Several options for applying exercise strategies have been described, along with patient handouts, by Bezherano et al. [385]; their adaptations to the research-based exercise protocols that typically comprise Buffalo Testing and home heart rate monitor, includes Buffalo Testing but without the home-based heart rate monitor and no in-office testing following a structured home-based program using a heart rate monitor. In general, early research suggests that the use of a structured program seems to be more efficacious than relying on patients to develop their own unmonitored program [454]. The recommendation that “*patients are encouraged to keep a symptom and exercise diary and return for re-evaluation every 1 to 2 weeks”* seems an appropriate visit frequency for patients engaging in a graduated aerobic exercise program to support a meaningful exercise intervention [385].

## 11. Nutrition Module

### 11.1. Neuroanatomical Principles: Nutrition and Concussion

The preceding sections regarding sleep, fatigue, and exercise are the core components that we prioritize in post-concussion rehabilitation with post-acute patients. The research base for the subsequent two modules discussed here (nutrition and relaxation) are less developed in the field of concussion. Nevertheless, the known neuroanatomical principles associated with these two additional areas present opportunities for optimizing recovery and are included in the treatment program of most patients we encounter.

The reason why nutrition is relevant in concussion rehabilitation relates to the following:

- The previous discussion on inflammation and the capacity for diet to be a powerful mediator of unhelpful inflammatory responses and other secondary injury cascades post-injury.
- Suboptimal nutrition’s capacity to dysregulate the homeostatic regulatory systems we have discussed in this article.
- The brain’s energy needs, which can be compromised by the injury-related impairment of energy generating systems in the brain.

A brief overview of some core physiology related to nutrition is presented below, followed by the association of these basic principles to these three factors.

#### 11.1.1. A Brief Overview of the Gut Microbiome

The human gut microbiome consists of 10 to 100 trillion microorganisms that reside in the gastrointestinal tract; these microorganisms impact the immune system, harvest energy from food, and impact a wide range of human diseases and behaviors [455,456]. We are beginning to understand what constitutes a healthy gut microbiome (eubiosis) and, conversely, how a compromised microbiome (dysbiosis) contributes to disease [456]. Research in recent decades has shed light on the importance of gut microbiome health for both psychological and physical wellbeing [457,458,459,460]. The gut microbiome can be highly variable between individuals, and the largest factor accounting for this variability is attributable to diet [456]. An acute change in diet can alter the microbial composition of the gut microbiome within 24 h [461].

To promote the health of the gut microbiome, nutrition research supports the utility of daily consumption of a wide variety of different colored fruits and vegetables—an approach sometimes referred to as “eat a rainbow.” The different colors found in fruits and vegetables reflect the relative abundance of one or more color-associated bioactive phytonutrient categories: carotenoids, flavonoids, betalains, and chlorophylls (see the systematic review of health effects of colorful bioactive foods in [462]). In addition to enhancing the gut microbiome composition, this nutrition approach provides the body with a variety of vitamins, minerals, and bioactive compounds known as phytonutrients. Phytonutrients are used by the body to enhance health, including factors highly relevant in concussion rehabilitation: reducing inflammation, supporting energy metabolism, and neuroprotective effects [68,463].

Unhealthy eating habits have been shown to influence the balance of intestinal microbiota of the gut microbiome, change blood–brain barrier permeability, and increase neuroinflammation [73,464]. Neuroinflammation is associated with a variety of cognitive and mood disorders in the general population [465]. Gut microbiome health can be further compromised following traumatic brain injury, secondary to the systemic stress and inflammation associated with injury and disruption to the optimal functioning of the gut–brain axis, which contributes to chronic neuroinflammation [461,466,467]. Efforts to restore a healthy gut microbiome following brain injury improve neurologic deficits in animal models [468,469]. Early studies in human mTBI populations identified shifts in gut microbiome populations that can leave people more vulnerable to systemic inflammation and symptoms associated with increased intestinal permeability [470,471].

#### 11.1.2. Nutrition, Brain Injury, and Neuroinflammation

Several authors have noted that suboptimal nutrition has the potential to hamper recovery post-brain injury. Animal TBI model research suggests that consumption of a “Western diet” (a diet high in calorie content, animal protein, refined sugars, refined carbohydrates, ultra-processed foods, and saturated fat, with inadequate amounts of fiber, fruits, and vegetables) has the potential to hamper recovery from TBI due to associated systemic inflammatory responses, neuroinflammation, and impaired neuronal homeostasis [68,397,472,473].

Secondary injury effects following mTBI include oxidative stress and neuroinflammation [474]. A Western diet can magnify these anomalies due to its impact on the increased release of reactive oxygen species, oxidative stress, and reduced synaptic plasticity [475]. In contrast to the Western diet, antioxidants and flavonoids from the consumption of vegetables and fruits have the potential to reduce neuroinflammation and oxidative stress in the brain [397,476,477].

#### 11.1.3. Nutrition, Brain Injury, and Regulatory Systems

In addition to diet’s impact on inflammation, dietary patterns also impact the autonomic and endocrine stress systems. In humans, there is a close relationship between insulin levels and activation of the sympathetic nervous system. Eating high-glycemic foods such as starches and sugars that spike insulin leads to a parallel increase in sympathetic nervous system activity and reduced efficiency of autonomic cardiac control [478,479].

Researchers have found markers of chronic overactivation of the sympathetic nervous system, decreased activation of the parasympathetic nervous system, and increased HPA axis activity and cortisol levels in humans and primates that consume a Western diet when compared with a Mediterranean-style diet [480,481,482]. Furthermore, stress resilience appears to be impacted by diet; primates fed a Mediterranean diet, when compared with those fed a Western diet, demonstrated lower sympathetic activity in response to stress, quicker heart rate response in the face of stress, more rapid recovery of heart rate following stress removal, and lower cortisol responses to the stressor [483]. In addition to the impact on cortisol levels of the HPA axis, additional hormonal anomalies are associated with various dietary patterns, including impacts on sex hormones, insulin sensitivity, and satiety hormones (see review in [484]). Below, we discuss the features of healthy diets such as the Mediterranean diet.

#### 11.1.4. Nutrition, Brain Injury, and Energy Systems

In the context of acute mTBI, some patients will experience inefficiencies in the energy-producing mitochondria of the brain at the very time the energy demands in the brain to promote homeostasis experience an acute surge [111,212,213,485,486,487]. In the acute phase, concussion causes ionic and metabolic changes that induce an increased need for glucose and other nutrients [488]. This is followed by a prolonged state of hypoglycolysis, which can leave the brain vulnerable to secondary injury cascades [489]. Research indicates that the Western diet can impair the functioning of mitochondria in the brain and interfere with neurochemicals involved in brain metabolism [475]. These findings highlight the importance of ensuring a quality and consistent nutrient supply to an organ with known energy synthesis disruption.

The importance of a quality nutrient supply following mTBI may be especially relevant when considering that the brain is a very energy-hungry organ. Glucose is the primary fuel for the human brain [270]. Approximately 20% of the circulating glucose gleaned from feeding is allocated to brain functioning under normal conditions, despite the brain weighing only approximately 2% of body mass [475,490,491].

#### 11.1.5. Dietary Patterns to Promote Health and Healing

In a recent review in *Nature Reviews Microbiology* [492], a number of whole-food diets (including Mediterranean, high-fiber, plant-based, and high-protein diets) were contrasted with the impact of the Western diet on the gut microbiome. Compared with diets based primarily on whole, unprocessed, or minimally processed foods, the Western diet was associated with a marked reduction in microbiome diversity (dysbiosis) and a surge of chronic systemic inflammation. Other authors have also commented on these contrasting effects of different dietary patterns on inflammation and the gut microbiome [473,493,494,495].

In contrast with the Western diet, the Mediterranean diet is one example of a highly regarded, healthy, and balanced diet [461]. It is distinguished by a beneficial fatty acid profile that is rich in both monounsaturated and polyunsaturated fatty acids from olive oil and fish. These types of oils have anti-inflammatory, antioxidant, and neuroprotective effects and tend to be more stable when heated compared with highly processed seed and vegetable oils [496,497,498,499]. The diet also includes high levels of polyphenols and flavonoids from a high intake of a variety of fruits and vegetables, which have the potential to reduce oxidative stress, and a relatively greater vegetable than animal protein intake. Specifically, the main constituents typically include olive oil, assorted fruits, vegetables, cereals, legumes, and nuts; moderate consumption of fish, poultry, and red wine; and a lower intake of dairy products, red meat, processed meat, and sweets. There is a well-established literature supporting the association of the Mediterranean diet with improved gut microbiome diversity and overall anti-inflammatory effects [493,500].

In summary, eating patterns associated with the Western diet have the potential to impair the health of the gut microbiome, increase systemic inflammation, increase neuroinflammation, increase circulating stress hormones, artificially elevate sympathetic nervous system activity, reduce parasympathetic activity, interfere with brain energy metabolism and mitochondrial functioning, and result in slower resolution of acute stress responses. In contrast, transitioning to dietary patterns that are dominated by a wide variety of unprocessed or minimally processed plants and minimally processed unsaturated fats has the potential to improve the efficiency of regulatory systems in the body. This presents an opportunity for patients recovering from concussion to reap the recovery benefits of transitioning to a more optimal nutrition pattern [501].

### 11.2. Evidence: Nutrition and Concussion

#### 11.2.1. Supplements in Concussion

A number of potential dietary supplements have been proposed as potentially beneficial in concussion recovery, based largely on animal model research. These include supplementation with omega 3 fatty acids, pinus radiata extract, melatonin, magnesium, probiotics, cerebrolysin, ginseng, creatine, and caffeine (see reviews [307,502]). How these findings translate to humans is largely untested in controlled trials, and caution in overapplication of the results is indicated due to differences in nutrient bioavailability and metabolism between humans and other species.

Several reviewers have evaluated the evidence base of supplement trials in human populations with a history of concussion, with an overall consensus that there is insufficient evidence to recommend specific supplements in routine clinical practice [307,502,503]. For example, Feinberg et al. [502] concludes, *“…none of the interventions represented in this review have yet garnered sufficient evidence to justify their use as first-line therapeutic interventions in a clinical setting. Importantly, only 60.0% of the studies assessed adverse reactions.”* (See also [504].)

Another reason suggesting caution when recommending supplements is that there is a possibility for negative impact on potentially helpful mechanisms from overutilization of supplementation. For example, the inflammatory response after concussion is very complex; some aspects of inflammation support an appropriate and helpful response to injury, but when inflammation becomes inappropriately elevated or chronic, it can be damaging to recovery. Before specific anti-inflammatory supplements can be recommended, science will need to advance to the point that it can identify the features of a helpful post-concussion inflammation response, identify at what point the transition to an unhelpful response occurs, and identify supplements or medication options that effectively intervene at various points post-injury. These conditions have not been satisfied, leading some to conclude that “*In some cases, treatments that reduce the inflammatory response will also hinder the brain’s intrinsic repair mechanisms*” [489] (see also [505]). Furthermore, caution is also advisable when considering that some research has found that supplements may contain variable doses of the supplement itself, include multiple compounds not approved for human consumption, and have the potential to interact with prescribed medications [506].

#### 11.2.2. Dietary Interventions in Concussion

To our knowledge, there are no controlled studies that have directly evaluated dietary patterns of interventions (such as Mediterranean vs. Western diets) in concussion populations. Some authors have proposed that ketogenic diets may have the potential to decrease the impact of brain injury by improving energy availability and metabolism [307,397,501]. However, no published studies of ketogenic diets in human concussion research were identified other than one single-arm feasibility trial with underwhelming results on computerized cognitive testing and symptom profile [507].

Most authors propose that reducing highly processed foods that are associated with inflammation and increasing whole foods is a reasonable post-injury approach for concussion. Monti et al. [307], for example, reviewed the available research regarding diet and supplements and concluded, “*Clinicians should first assess and intervene in the diet as a whole before considering nutritional supplements… The evidence supporting a specific diet or supplementation regimen to enhance neuroprotection or mitigate mTBI symptomology in humans is not yet strong enough to formulate clinical guidance; however, dietary supplementation with nutrients discussed in this article as having potential benefit in TBI (e.g., omega-3 fatty acids, creatine, and vitamin D) is generally safe when taken within recommended guidelines. Additionally, the DOD’s [USA Department of Defense] Warfighter Nutrition Guide recommends eating a diverse, high-quality diet that includes colorful whole foods rich in antioxidants, phytonutrients, omega-3 fatty acids, micronutrients, probiotics, and sufficient fiber to optimize long term health and performance…*”

### 11.3. Clinical Judgment: Nutrition and Concussion

Traditionally, rehabilitation professionals have not tended to include dietary recommendations as part of their practice, and some clinicians may feel this falls outside the scope of their practice. However, as research into the impact of diet on neural functioning has blossomed in recent decades, the importance of arming patients with this information has become increasingly apparent in many clinical fields. An example of this shift in one professional group comes in the form of a recent educational paper from the USA’s National Academy of Neuropsychology, where discussions of nutrition and other “lifestyle factors” such as exercise and social engagement are now encouraged as part of a comprehensive evaluation and intervention effort for neurologic conditions [506]. This sentiment is echoed by the USA National Athletic Trainers’ Association position statement on the management of concussion in sport: *“A patient with a concussion should be instructed to eat a well-balanced diet that is nutritious in quality and quantity and should drink fluids to stay hydrated”* [508].

Based on the balance of evidence reviewed above, we do not recommend a specific dietary plan to patients over and above the general guidelines that are reviewed below. A Mediterranean-style diet would appear to hold the most promise for general dietary guidelines, but this has not been formally tested for concussion recovery in humans.

In terms of supplements, typically we review the limitations of research in humans and allow patients to make their own decisions, especially if they have engaged in supplementation prior to injury. We would also highlight with patients that supplements are exactly that—to supplement a quality diet—rather than promote the illusion that supplements can compensate for a poor-quality, Western-style diet. If a patient recovering from mTBI were to take omega-3 supplements and then head to a fast-food restaurant for soda, fries, and a hamburger, the supplement is of questionable utility. The vast majority of the patients we see have suboptimal diets, and we encourage them to focus the bulk of their energy related to nutrition on the optimization of quality dietary nutrition as noted below.

#### 11.3.1. Nutrition Advice for Patients

It is appropriate to be honest with patients that currently there is not a body of well-designed medical research that directly studies the best nutrition plan for recovering from concussion. Despite this lack of specific research, clinicians can share that there is a lot of research that helps us understand how nutrition influences health in general and how nutrition influences the brain, inflammation, and the regulatory systems in the body. Basic information about brain fuel demands post-concussion, inflammation post-concussion, and the opportunities for manipulating the diet to manage these factors is shared with patients, and a handout documenting this information can be helpful to share with patients. The handout also includes guidance regarding the change process, which focuses on growth and supporting curiosity about foods rather than approaching change from a standpoint of self-criticism, shame, or other emotionally punishing perspectives. The following general dietary advice may be personalized to the individual patient and guided by a dietitian as needed:

- Consume a mix of the core macronutrients on a daily basis, and ideally, in each meal: carbohydrates, proteins, healthy fats, and fiber.
- Get the bulk of your calories from unprocessed vegetables and fruits. Try to eat a wide variety of different-colored vegetables. This is sometimes called the “eat a rainbow” approach.
- Minimize processed foods.
- Stay well hydrated. Avoid sweetened beverages, including fruit juices.

It has been our experience that patients become enthusiastic about dietary change when they understand the rationale behind the dietary recommendations above. Many patients have been told intermittently by their primary care providers and other physicians to lose weight or make heathy food choices for decades, and this advice has been less effective for many of them [509]. The focus of the dietary recommendations advocated for in this paper are not about weight loss, and for most patients we would never consider the patient’s weight as a pragmatic factor for discussion. Rather, the focus is on nutrition for brain optimization and recovery. An approach that supports patients’ curiosity and excitement (fueled with accurate information and open, non-judgmental discussion) is a potentially potent approach for clinicians who have the opportunity to meet with patients multiple times a month to support behavioral modifications that can help their recovery.

## 12. Relaxation and Behavioral Activation Module

In the previous modules, we have highlighted opportunities to stabilize autonomic, endocrine, and immune system functioning via optimization of sleep, fatigue management, exercise, and nutrition. The last module discussed in this paper alerts patients to opportunities for behavioral interventions that have known benefits to autonomic nervous system stability. These include (a) deep-breathing training, (b) behavioral activation/positive event scheduling, and (c) incorporating outdoor activities in nature. The anatomical principles, evidence in concussion studies, and clinical judgment insights associated with these three modalities are discussed in the following sections. The research base and historic use of these interventions in concussion rehabilitation is limited. Thus, the information regarding these modalities is briefer than the information covered in the previous modules.

### 12.1. Deep-Breathing Training

Slow-breathing training in the general population and in various clinical groups is associated with enhanced autonomic functioning, increased resilience to the endocrine cortisol stress response, and positive impact on the immune system [75,510,511,512,513,514].

Breathing training targeted at slowing the breathing rate at rest to approximately 6 breaths per minute increases parasympathetic activation and decreases sympathetic nervous system activity [515]. There are multiple modalities of breathing training practices that have the potential to enhance autonomic functioning, including yoga [516,517,518], progressive muscle relaxation [519], tai chi [520], mindfulness meditation [521,522,523], and resonant frequency breathing training [524,525]. Early research shows that cardiorespiratory anomalies correlate with symptoms following adolescent concussion [445].

The research evidence in support of using relaxation strategies in concussion rehabilitation is notable but in an early stage of development. We were able to identify only two case studies, one feasibility study, and one randomized controlled trial. The case studies comprised using progressive muscle relaxation in a multicomponent intervention with a 23-year-old soldier [526] and utilizing heart rate variability biofeedback in a 42-year-old athlete [527], both reporting positive outcomes. Usmani et al. [528] conducted a feasibility study of app-delivered progressive muscle relaxation with 49 mTBI subjects who had posttraumatic headache; overall, there was poor compliance with the app-based intervention. In a randomized controlled trial conducted in Taiwan, Lu et al. [529] evaluated the efficacy of heart rate variability biofeedback training (also known as resonant frequency breathing training) in an acute, adult, mTBI group comprising 41 completers randomized to either an educational control or a resonant frequency breathing training program. In contrast with those in the control group, those in the intervention group displayed improvements in neuropsychological tests of executive functioning, processing speed, and verbal memory; reduced post-concussive symptom scores; and reduced anxiety, depression, and irritability scores on questionnaires, with large effect sizes apparent at post-test 12 weeks after the initiation of the study.

In clinical practice, we advocate for patients implementing some form of relaxation practice into their recovery plan. The modality is often dependent on the patient’s previous experience with the modalities listed above. If they already have an active relaxation practice, we encourage them to continue with this or enhance the frequency to a daily practice. For those with no current practice, the most common intervention we initiate is resonant frequency breathing training, which can be performed using a biofeedback monitoring device in the clinic office. One of the advantages of biofeedback is that it can provide real-time information that the patient’s autonomic functioning is enhanced by the breathing training, which likely serves to bolster motivation for practice between sessions. In the absence of more personalized guidelines for a given patient, the patient may be instructed, for example, to engage in slow breathing at approximately 5 to 6 breaths per min (using a ticking clock/metronome or a freely available metronome app) for a minimum of 10 min, twice a day.

### 12.2. Behavioral Activation Therapy/Pleasant Event Scheduling

Behavioral activation (also known as positive/pleasant event scheduling) is a psychological treatment that is a core component of cognitive behavioral therapy [530]. In brief, the intervention supports patients to identify and schedule pleasant/fun activities into their week. Tracking the activities engaged in and the level of pleasure derived from these activities is often a core component [530]. Research with patients diagnosed with depression has shown that behavioral activation can be effectively implemented by providers who do not hold advanced degrees in psychological therapy and that behavioral activation is as efficacious as other psychological interventions (such as more comprehensive cognitive behavioral therapy programs) and is likely superior to antidepressant medications at mitigating depressive symptoms [531,532]. In addition to reducing the symptoms of depression, behavioral activation is an effective treatment for anxiety [533,534]. As previously discussed in this paper, people without a history of concussion who meet the criteria for anxiety and depressive disorders tend to report high levels of post-concussive-type symptoms; reducing mood- and anxiety-related symptoms (or the risk of developing them post-injury) has the potential to improve chances of a positive recovery post-mTBI.

The initial impetus to consider incorporating behavioral activation into our treatment program was inspired by animal research showing that enriched environments (cages with activities) are beneficial to recovery following mTBI in animal models [535]. Furthermore, behavioral activation in humans has been shown to increase positive mood states, which in turn are associated with improvements in autonomic functioning [536,537]. We concluded that, based on neuroanatomical principles, including behavioral activation as a component of our treatment would be protective against negative mood states such as anxiety and depression that mimic concussion symptoms, support the recovery of autonomic stability, increase relaxation opportunities, and promote exposure to the enriched environments that show promise for recovery in animal models of traumatic brain injury.

We were not able to identify any concussion studies that directly evaluated the efficacy of behavioral activation. However, the general movement away from rest and toward activity as an intervention following concussion is consistent with behavioral activation principles. Several authors have proposed that behavioral activation may be a relevant consideration in treating symptoms following concussion [353,538,539]. Early qualitative research suggests that the following adaptations should be considered in applying behavioral activation to patients with brain injury: balancing activities with available energy limitations, ensuring activities do not interfere with daily routines, support to cope with “failed activities,” and integrating activities with individual patient values such as family and social engagement [540].

In clinical practice, we typically implement specific behavioral activation strategies once patients have some experience tracking activities and energy in a log/diary (see the Fatigue Module). Integrating behavioral activation strategies by discussing with patients how to add “at least two activities that you think will be pleasant or fun each week” into their planned activities can merge relatively seamlessly with the activity tracking in the fatigue management section. Patients can add a “pleasure level” rating on their log next to activities they completed that they anticipated would be pleasant. Assigning patients to plan to have some fun is typically well received and is often a welcome respite from problem-focused intervention strategies.

Behavioral activation also supports patients to balance efforts to improve functioning in the rehabilitation areas of (a) work or school, (b) home and family, and (c) social and recreational pursuits. We have found that imbalances in overprioritizing work/school increases at the expense of home/family and recreational/social activities is rarely sustainable. For example, the patient that aggressively increases work hours during a reintegration trial and returns home depleted and unable to effectively manage home roles or regulate emotions in the family setting, with no activities in the week that they are looking forward to, will rarely escape gradual deterioration on all fronts.

#### 12.2.1. Comment on Nature-Based Behavioral Activation

Theorists in the 1980s and 1990s proposed that exposure to nature (i.e., non-urbanized settings) could have beneficial effects on attention and stress reduction [541,542]. While some researchers have focused on delivering rehabilitation interventions in natural environments such as forests, other research has attempted to clarify the physiologic impact of being immersed in nature as a behavioral activity (in some settings this is referred to as “forest bathing”).

Thus far, the current evidence supporting implementing rehabilitation therapies in a nature setting for patients with concussion is underwhelming. Vibholm et al. [543] conducted a scoping review of nature-based rehabilitation for patients with a history of acquired brain injury. The two forms of nature-based rehabilitation were categorized as either social/therapeutic horticulture (gardening in a group setting) or delivering rehabilitation interventions (such as education and mindfulness activities) in a pleasant natural environment. We were able to identify only two case series that applied these principles to patients with a history of concussion; only modest reductions in the primary outcome measure of fatigue were noted post-intervention [544,545].

While we await more research about the potential benefits of concussion rehabilitation efforts delivered in nature settings, an opportunity exists to encourage patients to take advantage of the psychological and physical benefits of immersion in pleasant natural environments. For example, Aras et al. [546] contrasted the physical effects of either walking on a woodland path or in an urban environment in the general adult population. Participants experienced higher levels of heart rate variability (suggesting improved autonomic functioning) and lower cortisol levels in the natural environment when compared with the urban environment. There is a growing body of research that supports the conclusion that increasing exposure to activities in nature has benefits for physical and emotional health [547,548,549,550,551,552,553,554,555,556,557,558].

We were not able to identify any studies that explored the impact of behavioral activation activities in nature with individuals recovering from concussion. Despite the lack of direct evidence, based on known physiologic impact in other groups, it is reasonable to encourage nature-based activities as part of a behavioral activation plan with these patients. In general practice, we have found discussions with mTBI patients regarding their previous positive experiences in nature, which activities they are drawn to re-engage in, and what specific plans they can make for engaging in nature-based activities are well received and appear useful to treatment progress. We highlight the general caution that supporting patients to select activities that minimize the risk of further injury is an important consideration.

## 13. Synthesis and Conclusions

In the previous sections, we have highlighted opportunities for intervention with patients who experience persisting symptoms following concussion injuries. It has been our experience that implementing this foundation program—incorporating sleep stabilization, fatigue management, physical exercise, nutrition optimization, and relaxation/behavioral activation strategies—is a potent combination that leads to reduced symptoms and improved functioning in the patients we have served.

### 13.1. Comment on Potential Iatrogenic Factors

The program described above targets the biological processes that underly a wide range of post-concussive complaints. Improvements gleaned by implementation of the modules described in this article tend to be global (across the spectrum of post-concussive complaints), including the physical symptoms, cognitive symptoms, emotional symptoms, and sleep symptoms associated with concussion.

What many of our patients encounter in seeking support for their persisting post-concussive complaints prior to entering our clinics is a symptom-focused approach. This is important to note, as many patients referred to our clinics have a haphazard array of treatments already in place for many of the symptoms they present with. They may be seeing a headache specialist; physical therapy for orthopedic issues; speech therapy for attention and memory difficulties; vision therapy; vestibular therapy; and various chiropractic, massage therapy, and psychological interventions, all concurrently. In the context of increasing specialization in healthcare delivery, this pattern appears more commonly over time.

The overapplication of the “engage with multiple specialists to treat the individual symptoms approach” has the potential to be problematic for some patients. Most patients we encounter struggle with fatigue and reduced functioning in home and occupational/academic roles. Many are desperate to engage in treatment to improve and are eager to engage with specialists who have expertise in their many problems. However, the patient who is struggling to catch up in school, increase work hours, care for their children’s needs, or deal with any number of other demands in their daily life may have limited resources (emotional, energy, time, financial) to engage in multiple therapies at the same time. Gauging the resources a patient has to engage in time- and energy-demanding treatments is an important factor for clinicians to consider and discuss with patients.

In some cases, “overtreated” patients become so depleted that their post-concussive complaints can increase over time or be unnecessarily maintained by the overexertion associated with compliance with multiple treatments (many of which include home-based assignments between treatment appointments). This deterioration is an iatrogenic risk factor (iatrogenic is a term that refers to the potential adverse impact from the treatment itself).

As patients engage with multiple healthcare providers, they may also receive multiple and potentially conflicting messages about the underlying cause of their symptoms following concussion, recommended treatments, and expectations for recovery. Some patients may hear that they have brain damage or that they should avoid any activities that could possibly exacerbate their symptoms. This could affect the patient’s expectations for recovery and could contribute to avoidance of activities that could promote recovery. The *VA/DoD Clinical Practice Guideline for the Management of Concussion-Mild Traumatic Brain Injury* [559] states, *“Some presenting symptoms may be attributed to the mTBI event by both providers and patients, even though the contribution of the original event to current symptoms is uncertain. This can place the patient into a category in which all of his or her symptoms are considered ‘mTBI symptoms.’ This attribution, and potential misattribution, of symptoms to mTBI can potentially place the patient at risk. When such a patient becomes ‘a TBI patient,’ providers may continue to view all of his or her symptoms through that prism. Unfortunately, some of the very programs that are intended to help patients with a history of mTBI may have the unintended consequence of reinforcing the concept that all of his or her symptoms are mTBI-related. When this happens, the patient may consider himself/herself as a ‘lifelong’ mTBI patient…”*

In addition to potential iatrogenic increases in post-concussive-type complaints and reductions in functioning, another potential risk of overtreatment is that their potential for gains from valuable treatments is attenuated and time and resources are wasted. For example, a patient who is sleeping well, eating well, engaged in physical activity, and balanced in managing their fatigue is more likely to benefit from specialized interventions (such as those listed below) than one who enters treatment depleted. There are some notable exceptions where early concurrent referral for treatment of comorbidities is warranted. These were previously discussed (PTSD, cervical strain/pain conditions, vertigo). But in most cases, improvements in a wide array of presenting concerns will result from the implementation of the foundation program discussed in this paper.

We sometimes discuss iatrogenic risks with patients and contact other providers to explain their current depleted state and negotiate reductions in the frequency of appointments or postpone ongoing implementation of some interventions. We advocate for a general goal that most patients engage in no more than two rehabilitation appointments each week, with one of those appointments to focus on the modules discussed in this paper.

### 13.2. Next Steps

Once these modules are in place, many patients will find that they are improving in symptom targets, and tapering of sessions or ending treatment following implementation of a home-based maintenance program can be considered. In cases where specific symptoms persist, referral for targeted interventions for clinically relevant residuals can be considered. Such interventions may include the following:

- Cervical, vestibular, and vestibulo-ocular physical therapy interventions [153,560].
- Cognitive rehabilitation strategies [418,561].
- Vision therapy [562,563,564], although more robust evidence is needed regarding the effectiveness of vision therapy in mTBI rehabilitation [565].
- Posttraumatic headache interventions [566].
- Pituitary hormone screening [99,567].

### 13.3. Weaknesses and Strengths

The treatment model presented in this paper has several limitations:

- While several of the components of the treatment program have been the subject of treatment research exploration in populations recovering from concussion, the overall framework has not yet been evaluated in research trials but is based on neuroanatomic principles and clinical experiences that are suggestive of value. While this is to be somewhat expected based on the current state of concussion research in general, it remains a significant limitation.
- The model requires most clinicians to broaden their scope of practice and upskill in some areas. Developing skills in behavioral sleep interventions, exercise interventions, fatigue management, nutrition interventions, behavioral activation strategies, and relaxation protocols is achievable and within the capacity of most clinicians. However, it does require time and effort on the part of the clinician to organize resources, understand the material, and develop a plan for implementation with patients. Some clinicians are excited by the prospect of learning new skills and some are not, preferring to focus their continuing education efforts on narrower specialties. Both are legitimate choices. It is our impression that the initial foray into intervention with patients with persisting complaints requires clinicians with a broader rather than a narrower skill base.
- In protracted cases (such as symptoms present for 6 months or more), unless the treating clinician has the opportunity to meet with the patient regularly at first (ideally weekly, at a minimum fortnightly), the intervention described in this paper will likely be less effective. Some clinical practices are overwhelmed, and individual clinicians may struggle to shift systemic practices in the clinics where they work to allow for such regular contact. In cases where intervention cannot be this frequent, we would discourage the attempt at the program described in this paper. An inadequate, ineffectual dose has the potential to be demoralizing to a patient who is invested in complying with recommended interventions but is making little progress. In settings where frequent visits are prohibitive, clinicians can consider educating and engaging other clinicians in the treatment process, for example, by providing a written summary of modules to a psychological therapist that the patient is already seeing regularly to determine if that clinician is open to integrating some of the modules into that setting.

The treatment model we have presented in this paper has several strengths:

- It can be implemented by a wide variety of rehabilitation professionals with only modest training and supervision needs.
- It is relatively lean and cost-effective in terms of (a) a low need for specialized equipment and (b) clinical intervention time. Most patients can be served by one or two clinicians to implement this program rather than an entire multidisciplinary team (see comments regarding iatrogenic risks above).
- There is a clear rationale for the modules based on the neurophysiology of concussion and human physiology in general. Sharing this rationale with patients tends to increase engagement and compliance.
- It is respectful of both the limitations and strengths in the research and provides a balanced approach based on the available evidence.
- The strategies in the program (while particularly relevant for concussion patients) are beneficial for all humans across the developmental lifespan, and this presents opportunities to involve significant others in the treatment process in an effort to create a family culture that values these “lifestyle interventions.”
- The interventions are beneficial for multiple other health and emotional conditions and represent a set of behaviors that, in most cases, would benefit patients if they were continued for life, even after concussion symptoms resolve.

In conclusion, there are large numbers of people who struggle with debilitating symptoms following concussion injuries. We have presented a foundation intervention program that has the potential to mitigate persisting difficulties in this population. We hope that this information will benefit individual patients and promote the discussion of treatment opportunities in clinical systems. We anticipate that ongoing research will further inform developments in the field. As such, this paper is not a terminal position, but rather, just one voice in the ongoing evolution of ideas targeting the relief of suffering following concussion injuries.

## Data Availability

No new data were created or analyzed in this study. Data sharing is not applicable to this article.
